# Implementing Taiwan National Health Insurance Quality Indicators as Clinical Quality Language on Fast Healthcare Interoperability Resources: Development and Feasibility Study

**DOI:** 10.2196/108093

**Published:** 2026-10-15

**Authors:** Meng-Chi Chen, Yu-Cheng Pei, Kun-Ju Lin, Shao-Chi Lin, Yueh-Peng Chen, Yu-Chun Lin

**Affiliations:** 1 Department of Dentistry Chang Gung Memorial Hospital at Taipei Taipei Taiwan; 2 Department of Physical Medicine and Rehabilitation Chang Gung Memorial Hospital at Linkou Taoyuan Taiwan; 3 Master of Science Degree Program in Innovation for Smart Medicine Chang Gung University Taoyuan Taiwan; 4 School of Medicine, College of Medicine Chang Gung University Taoyuan Taiwan; 5 Department of Nuclear Medicine and Molecular Imaging Center Chang Gung Memorial Hospital at Linkou Taoyuan Taiwan; 6 Department of Medical Imaging and Radiological Sciences Chang Gung University Taoyuan Taiwan; 7 Department of Management Science National Yang Ming Chiao Tung University Hsinchu Taiwan; 8 Department of Physical Medicine and Rehabilitation New Taipei Municipal TuCheng Hospital, Chang Gung Memorial Hospital New Taipei City Taiwan; 9 Center for Artificial Intelligence in Medicine Chang Gung Memorial Hospital at Linkou Taoyuan Taiwan; 10 Department of Medical Imaging and Intervention Chang Gung Memorial Hospital at Linkou Taoyuan Taiwan

**Keywords:** Clinical Quality Language, cross-institution, data provenance, electronic clinical quality measures, FHIR, Fast Healthcare Interoperability Resources, medication safety, National Health Insurance, reproducibility

## Abstract

**Background:**

Clinical Quality Language (CQL) and Fast Healthcare Interoperability Resources (FHIR) support executable electronic clinical quality measures. Translating national claims–based indicators into CQL remains challenging because terminology, exclusions, and data semantics require adaptation. To our knowledge, Taiwan National Health Insurance (NHI) medical-center indicators have not previously been represented as an executable CQL library.

**Objective:**

This study aimed to develop CQL libraries derived from 39 Taiwan NHI medical-center quality indicator codes and evaluate technical executability and computational consistency using synthetic data and real-world cross-institution medication records.

**Methods:**

We implemented 39 CQL libraries across 6 principal FHIR R4 clinical resources using 21 data elements, with documented adaptations where official requirements could not be represented. Compiled Expression Logical Model was executed against author-defined synthetic fixtures. In a retrospective 2-campus study, a separately coded Python implementation and the CQL pipeline computed aggregate numerators and denominators for 20 indicators from the same deidentified records, represented as a long table and FHIR resources, respectively.

**Results:**

All 39 CQL libraries compiled and executed against the predefined synthetic fixtures, and their outputs matched the author-defined expected results (39/39 libraries; 136/136 early-refill grace-period control groups). The real-world dataset included 88 patients (Taipei: n=65; Linkou: n=23), 1597 medication records, and 630 visits from January 1 to June 2, 2026. Across the 20 medication-based indicators, the 2 pipelines produced identical aggregate numerators and denominators (20/20), and 7 of the 8 cross-institution drug-class overlap indicators had nonzero numerators. All 2317 FHIR resources passed base FHIR R4 structural validation, and Anatomical Therapeutic Chemical mapping coverage was 98.3% (1570/1597 records).

**Conclusions:**

The implemented NHI-derived indicator logic was executable and computationally consistent across synthetic testing and aggregate real-world comparisons. These findings do not establish complete fidelity to official NHI specifications. Further evaluation requires expert semantic review, patient-level testing, and execution across independent CQL/FHIR environments.

## Introduction

Clinical Quality Language (CQL), paired with Health Level Seven (HL7) Fast Healthcare Interoperability Resources (FHIR), supports reusable clinical logic and a standard data model for electronic quality measurement [1,2]. Specifications can be compiled to the Expression Logical Model (ELM) and executed on systems with compatible FHIR profiles, terminology mappings, and CQL engines. Despite increasing adoption, evidence of validated production deployment remains limited [3,4].

Recent work has addressed scalable CQL execution through a high-performance FHIR server with an embedded engine [5] and a large language model (LLM)-in-the-loop approach [6]. However, evaluations executing a broad national indicator library over real FHIR data and comparing results with a separately coded implementation remain rare.

Portability also requires adaptation to local electronic health records and workflows [7-9]. Translating narrative indicators into executable logic can introduce semantic differences, requiring clinical review and version control [9]. Similar difficulties have been reported when translating World Health Organization (WHO) recommendations into computer-readable FHIR artifacts [10].

Validation also requires an appropriate reference standard. Even high-performing digital quality measures are typically benchmarked against manual chart review, and inconsistent coding across systems directly erodes electronic clinical quality measure accuracy [11,12]. Establishing agreement between an automated computation and an independently coded reference implementation therefore remains a necessary intermediate step, though computational agreement does not substitute for clinical adjudication.

Taiwan’s single-payer National Health Insurance (NHI) program and national cloud query service provide cross-institution medication records with identifiers that distinguish prescribing institutions. Taiwan has also developed national FHIR infrastructure, including the Taiwan Health Application Space (THAS), a national SMART (Substitutable Medical Applications, Reusable Technologies)-on-FHIR application platform [13-15]. Study records came from the NHI cloud query service, not THAS. A search of PubMed, Scopus, and the HL7 CQL implementer registry, conducted through June 2026, identified no previous implementation of the official NHI medical-center indicators as executable CQL or computation on real FHIR data.

We developed 39 executable CQL libraries derived from NHI medical-center quality indicator codes and implemented them across 6 principal FHIR R4 clinical resources using 21 data elements. We also documented 3 implementation issues encountered during deployment, added execution traceability linking indicator results to source patients, rule versions, and query parameters, and performed aggregate differential testing for 20 computable indicators in an 88-patient, 2-campus cohort. This study evaluates the technical execution and computational consistency of the implemented logic; validation against the complete official NHI specifications will require additional semantic review and external testing.

## Methods

### Study Design and Overview

This study comprised 2 parts. The first was platform development, with technical execution and internal computational verification against author-defined synthetic fixtures in a local FHIR R4 research environment. The second was retrospective, 2-campus aggregate differential testing of outpatient and medication-safety indicators at Taipei and Linkou Chang Gung Memorial Hospitals, using deidentified NHI outpatient claims combined with cross-institution medication records from the national NHI cloud query service. Table 1 gives the indicator scope at each stage and the reason for each reduction. This study is reported in accordance with the Statement on Reporting of Evaluation Studies in Health Informatics (STARE-HI) [16]; the completed checklist is provided in Multimedia Appendix 1. Because cohort-level bulk extraction was unavailable, data were exported electronically, patient by patient, into a structured medication long table under a predefined specification, with no values manually transcribed. A separately coded Python reference implementation (pipeline A) computed indicators from the long table, and the platform CQL engine (pipeline B) computed the same study-operationalized definitions from those records represented as FHIR R4 resources.

**Table 1:** Indicator scope at each stage of the study.

Study stage	Indicators, n	Scope	Reason for reduction
Developed as executable CQL^a^ prototypes	39	39 NHI^b^ indicator codes across 5 categories; adaptations documented	Not applicable
Technically and computationally verified on synthetic data	39	Author-defined known-answer FHIR^c^ R4 fixtures with branch coverage	Not applicable
Targeted for initial real-world testing	23	Outpatient and medication-safety indicators (01, 02, 04-08, 03-1 to 03-16)	16 inpatient, surgical, and outcome indicators require inpatient and procedure data not available for real patients
Computable from the current export	20	Indicators 01, 02, 04, 05, and 03-1 to 03-16	Indicators 06 to 08 additionally require diagnosis (Condition), laboratory (Observation), and specific encounter-type data not present in the current export
Aggregate N/D^d^ concordance in differential testing	20	Pipeline A (Python reference) versus pipeline B (platform CQL engine)	Not applicable

^a^CQL: Clinical Quality Language.

^b^NHI: National Health Insurance.

^c^FHIR: Fast Healthcare Interoperability Resources.

^d^N/D: numerator/denominator.

### Evaluation Framework and Claim Boundaries

We defined 3 levels of evidence and evaluated the study against them: technical executability, internal computational verification, and aggregate differential concordance. Table 2 states each definition and the levels addressed in this study. These 3 terms are used consistently throughout. Levels beyond these 3—semantic fidelity to the complete official specifications, patient- and event-level agreement, external agreement with official NHI-calculated results, and cross-engine or cross-institution portability—define the subsequent validation phases and are set out in Table 2.

**Table 2:** Levels of evidence defined for this study.

Evidence level	Definition	Addressed in this study
Technical executability	Successful CQL^a^-to-ELM^b^ translation and execution without fatal errors	Yes; 39 libraries
Internal computational verification	Agreement with predefined expected results from deterministic synthetic fixtures	Yes; 39 libraries and 136 control groups
Aggregate differential concordance	Agreement between the aggregate outputs of 2 separately coded pipelines on the same source records	Yes; 20 indicators
Semantic fidelity	Rule-level correspondence with the complete official NHI^c^ technical definitions	Next phase
Patient- and event-level concordance	Agreement at the level of individual patients and prescribing events	Next phase
External and cross-engine portability	Execution on an external end point, a second CQL engine, or TW Core^d^ profiles	Next phase

^a^CQL: Clinical Quality Language.

^b^ELM: Expression Logical Model.

^c^NHI: National Health Insurance.

^d^TW Core: Taiwan Core.

### Platform Architecture

A browser front end communicates with a Node.js and Express back end, which compiles CQL to ELM JSON and executes it using cql-execution and cql-exec-fhir. Research computation used a local Health Level Seven Application Programming Interface (HAPI) FHIR Server, FHIR R4 version 4.0.1, accessed through Representational State Transfer without application-level authentication within the isolated environment. The separate public demonstration queried only the THAS CapabilityStatement end point. Figure 1 shows the architecture; component versions are listed in Multimedia Appendix 2.

**Figure 1:**
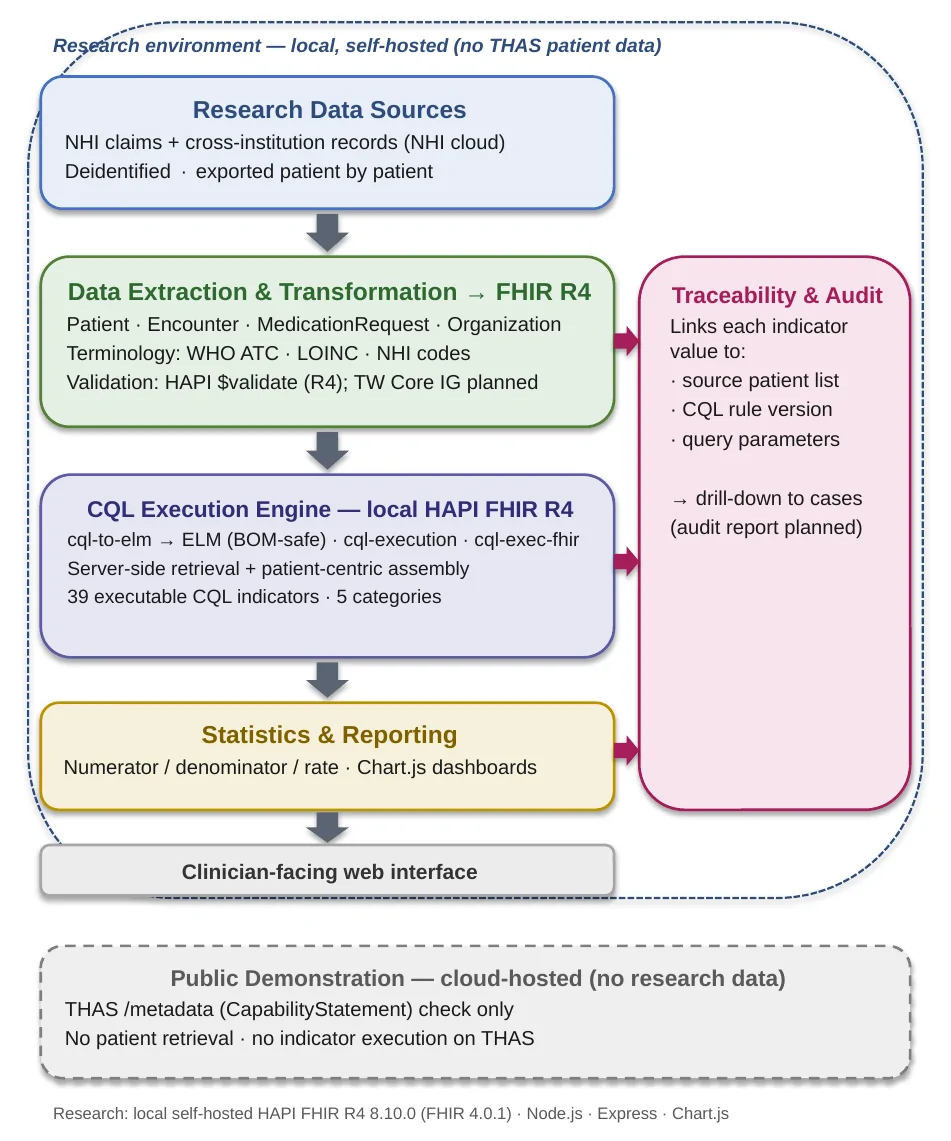
Platform architecture and research execution path. Research data flow from National Health Insurance (NHI) outpatient claims and cross-institution medication records through Fast Healthcare Interoperability Resources (FHIR) R4 transformation, Clinical Quality Language (CQL) engine execution, and source-linked reporting. All research data were processed in a local, self-hosted Health Level Seven Application Programming Interface (HAPI) FHIR R4 environment; the public demonstration was cloud-hosted and contained no research patient data. ATC: Anatomical Therapeutic Chemical; LOINC: Logical Observation Identifiers Names and Codes; THAS: Taiwan Health Application Space; TW Core: Taiwan Core; WHO: World Health Organization.

### CQL Indicator Library

We developed 39 executable CQL libraries derived from NHI medical-center quality indicator codes across 5 categories: medication safety (n=19 indicator codes), outpatient quality (n=4), inpatient quality (n=2), surgical quality (n=11), and outcome quality (n=3). Indicator codes correspond to the official codes published under the NHI hospital global-budget quality information disclosure.

Terminology integration uses WHO Anatomical Therapeutic Chemical (ATC) codes for medications, applied according to the established ATC and defined daily dose (DDD) methodology for classifying and quantifying medicine use in routine administrative data [17]. Logical Observation Identifiers Names and Codes (LOINC) is used for laboratory tests, together with FHIR R4 native status value sets. Injection and antibiotic use are identified from the eighth digit of the NHI order code. The medication-safety and outpatient indicators (01, 02, 03-1 to 03-16, 04, 05), which are the only indicators evaluated on real-world data here, require neither RxNorm, Systematized Nomenclature of Medicine Clinical Terms (SNOMED CT), *ICD* (*International Statistical Classification of Diseases*)-to-SNOMED CT mapping, nor Unified Code for Units of Measure; where a diagnosis is required (eg, indicators 16 and 17), *ICD-10-CM* (*International Classification of Diseases, Tenth Revision, Clinical Modification*) codes are used directly.

The implemented logic includes adaptations to official definitions: visit-based denominators for indicators 01 and 02, broader ATC scopes for some overlap indicators, incomplete claims-specific exclusions, and SNOMED CT proxies for selected synthetic-only surgical indicators. Outputs therefore represent study-operationalized indicators rather than official NHI rates. Multimedia Appendix 3 documents requirements, mappings, adaptations, test coverage, and gaps against the official specifications [18].

### FHIR Data Model and Field Footprint

The platform uses 6 principal clinical FHIR R4 resource types (Patient, Encounter, MedicationRequest, Observation, Condition, and Procedure), with Organization as a supporting resource for participating-site provenance. The real-world bundle contained 2 Organization resources for the 2 participating campuses; cross-institution distinctions additionally relied on the source NHI medical-institution identifier retained with each record. Across the 39 libraries, the implementation activated 21 of 190 enumerated standard data elements. This describes the observed field footprint, not the full cost of adoption, which also includes terminology, profiles, extensions, version governance, and temporal semantics (Multimedia Appendix 3).

### Implementation Notes: 3 Deployment-Layer Findings

Building and operating the platform surfaced 3 deployment-layer issues that materially affect whether a CQL toolchain can be operated reproducibly, each reported as an implementation observation rather than a solved infrastructure problem. First, CQL files saved as UTF-8 with a byte-order mark caused the cql-to-elm lexer to emit no ELM while the build still reported success, so the fault appeared only as a single missing file; we now write all CQL without a byte-order mark and fail the build on a precompilation check. Second, and architecturally, we execute CQL/ELM in the back end rather than from the browser: it performs paged FHIR queries, regroups resources per patient into FHIR collection bundles, and executes the compiled ELM. This centralizes execution and paging and keeps the client independent of end point–specific cross-origin resource sharing (CORS) configuration; it was a design choice rather than a workaround, and we report it because per-patient bundle assembly is a prerequisite for CQL execution that FHIR servers do not provide natively. Third, in the absence of shared verification fixtures, we built an answer-first test set whose expected-result manifest specifies the patients, numerators, denominators, and rates for the 39 libraries, instantiated by a deterministic synthetic dataset of 1473 patients across 4 simulated hospital Organizations [19]. Multimedia Appendix 2 documents the compiler defect and its controlled reproduction in full.

### Cross-Institution Medication Overlap Algorithm

#### Medication-Overlap Computation

For the cross-institution medication-overlap indicators (codes 03-9 to 03-16), the platform compares drugs at the WHO ATC class level, computes overlapping prescription days for same-class drugs, and assigns a risk level from cumulative dosing expressed as WHO DDDs. The institutional boundary is the distinct NHI medical-institution identifier carried by the source record and retained in the FHIR representation. When the same patient has same-class drugs under 2 or more distinct source identifiers, the overlapping drug-days are eligible to contribute to the cross-institution numerator after the early-refill grace-period rule is applied (Figure 2).

**Figure 2:**
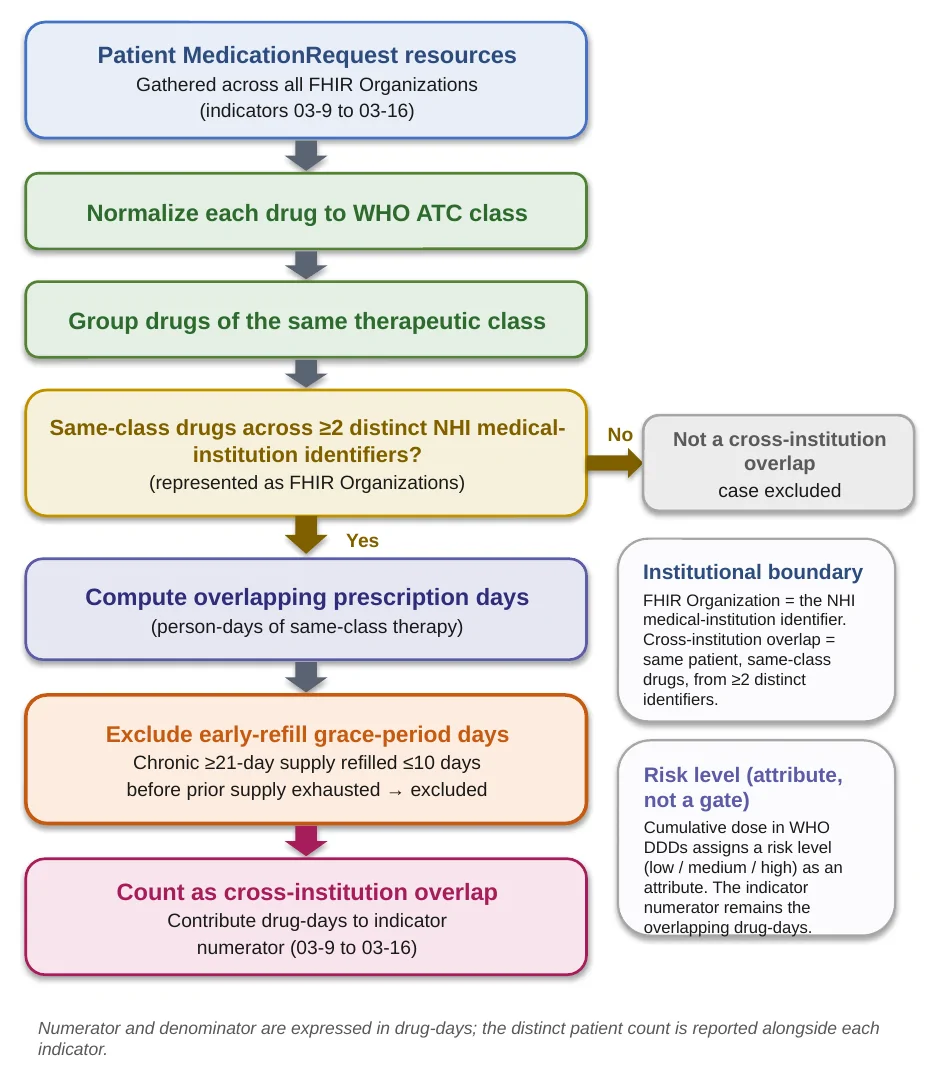
Cross-institution medication-overlap algorithm. MedicationRequest resources retain the source NHI medical-institution identifier, are normalized to the World Health Organization (WHO) Anatomical Therapeutic Chemical (ATC) class level, and are compared across distinct identifiers. Same-class overlapping prescription days contribute to indicators 03-9 to 03-16 after exclusion of early-refill grace-period days. A record that does not cross an institutional boundary is not included in the cross-institution numerator; it is not necessarily excluded from other denominator calculations. Cumulative dose in WHO-defined daily doses (DDDs) assigns a risk attribute, while the numerator remains the overlapping drug-days. FHIR: Fast Healthcare Interoperability Resources; NHI: National Health Insurance.

#### Institutional Boundaries in the Real-World Data

Each record retains its prescribing or dispensing institution identifier. The 2 Organization resources represent the participating campuses; identifiers from external institutions remain source provenance. Cross-institution overlap therefore includes institutions outside Chang Gung without requiring an Organization resource for each external identifier.

### Execution Traceability

The platform records execution metadata linking computed indicator values to contributing patient lists, the CQL rule version, and query parameters, supporting preliminary drill-down and source tracing.

### Ethical Considerations

This study was approved under expedited review by the Chang Gung Medical Foundation Institutional Review Board (IRB number 202601223B0), which granted a waiver of participants’ informed consent. The study is retrospective and uses existing deidentified outpatient claims and medication records, with no participant contact and no applicable compensation.

Data transmitted outside the hospital, including cross-institution records returned by the national cloud query service, were deidentified using an approach informed by the 18 Health Insurance Portability and Accountability Act (HIPAA) Safe Harbor identifier categories and adapted to the Taiwanese legal and research context. The analysis reads only deidentified fields: visit sequence number, visit year-month, department code, the 10-digit NHI drug code, the ATC code, drug items per visit, the medical-institution identifier used only to distinguish institutions, and case category. Direct identifiers such as name, national identification number, medical-record number, and date of birth are not read.

### Evaluation 1: Synthetic Technical and Computational Verification

To assess technical executability and internal computational consistency, we constructed the purpose-built synthetic dataset described under Finding 3: 1473 patients across FHIR R4 transaction bundles (simulated service period May 1 to June 20, 2026), with branch-coverage allocation for every indicator and 4 simulated hospital Organizations enabling same-institution (03-1 to 03-8) and cross-institution (03-9 to 03-16) overlap scenarios. For the 16 medication-overlap indicators, it additionally encodes 136 control groups isolating the chronic-prescription (≥21-day supply) early-refill grace-period rule. Agreement with these author-defined fixtures evaluates the implemented study logic and does not establish fidelity to every official NHI requirement.

Verification was performed locally on the self-hosted HAPI FHIR server, with each indicator computed through the compiled-ELM pathway. Because the synthetic data were purpose-built known-answer fixtures, they cannot be interpreted as an epidemiological sample.

### Evaluation 2: Initial Real-World Differential Testing

#### Study Cohort and Evaluation Design

We designed a 4-stage evaluation of the 23 outpatient and medication-safety indicators listed in Table 1: the 7 visit-level outpatient and prescribing indicators (01, 02, 04, 05, 06, 07, and 08), the 8 same-institution medication-overlap indicators (03-1 to 03-8), and the 8 cross-institution medication-overlap indicators (03-9 to 03-16). The final analytic cohort comprised 88 patients across Taipei and Linkou Chang Gung Memorial Hospitals and was a convenience sample. Because records had to be exported patient by patient under the approved specification rather than extracted in bulk, patients were selected pragmatically from those attending the 2 campuses during the study window, without a prespecified sampling frame or quota, so the resulting campus distribution was not predetermined. No inclusion or exclusion criteria were applied beyond the availability of an exportable outpatient medication record, and no patient was excluded after selection because of incomplete data. The exported medication records supported indicators 01, 02, 04, 05, and 03-1 to 03-16; indicators 06 to 08 additionally require diagnosis (Condition), laboratory (Observation), and specific encounter-type data not present in the medication-only export.

Evaluation comprised 4 stages. The primary comparison was prespecified at the aggregate indicator level; patient- and event-level concordance was not assessed.

#### Stage A: Coarse-Grained ETL Validation (Resource-Structure Level)

The converted Patient, Encounter, MedicationRequest, and Organization resources were checked with the HAPI FHIR $validate operation against base FHIR R4 (4.0.1); per-resource pass rates were computed and issues classified by severity. Taiwan Core (TW Core) profile validation is planned for the subsequent interoperability phase.

#### Stage B: Terminology Assessment

We calculated NHI-to-ATC mapping coverage, listed unmapped codes and their frequencies, and assessed mappings for the medication records used by the platform.

#### Stage C: Platform Batch Computation

The platform CQL engine loaded the converted FHIR bundles and computed all supported indicators through the compiled-ELM pathway, recording numerator, denominator, rate, execution path, and per-call response time. For the 88-patient cohort, this computation constitutes pipeline B.

Stage D: differential testing. The independently coded Python reference pipeline (A) and CQL pipeline (B) computed the same operationalized definitions from the long table and corresponding FHIR resources, respectively. We compared aggregate numerators and denominators; interpretation of this agreement is addressed under Limitations.

### Execution-Time Measurement

Execution time was recorded at 2 levels: end-to-end response time at the deployed HTTP service for each of the 20 indicator calls on the 88-patient cohort, and engine-module instrumentation attributing elapsed time to FHIR resource retrieval, per-patient bundle assembly, and ELM evaluation. All timings were obtained from a single unoptimized research environment without caching, connection pooling, or parallel execution, and were summarized descriptively. The instrumentation protocol and the full per-indicator measurements are reported in Multimedia Appendix 2.

### Analysis

Outcomes were the numerator, denominator, and rate of each indicator, computed independently by the 2 pipelines. The unit of analysis differed by indicator family: medication-overlap indicators (03-1 to 03-16) used drug-days, defined as person-days of overlapping versus total prescribed therapy, with the contributing distinct patient count reported alongside, while the outpatient prescribing indicators (01, 02, 04, and 05) used visits, a visit being the combination of patient, medical-institution identifier, and start date. Rates were the numerator divided by the denominator over the study period.

Aggregate concordance was defined as exact equality of both numerator and denominator for a given indicator and summarized as the number of concordant indicators over the 20 evaluated. Because these 20 constituted the complete set computable from the current export rather than a random sample, concordance is reported as a proportion of that fixed set without a CI. Base FHIR R4 structural conformance, NHI-to-ATC mapping coverage, and execution times were summarized descriptively.

## Results

### Indicator Library and Maturity

All 39 libraries compiled and executed with author-defined numerators, denominators, and rates. Adaptations and known-answer fixtures are documented in Multimedia Appendices 3 and 4.

### Deployment-Layer Findings: Operational Verification

The 3 deployment-layer findings were assessed operationally. All CQL sources were converted to UTF-8 without a byte-order mark, and a precompilation check was added; 0 of 51 files retained a mark, and all 39 libraries produced ELM (39/39), while deliberately reintroducing a mark reproduced the silent failure and removing it restored byte-identical output (Multimedia Appendix 2). The back end assembled 88 of 88 per-patient FHIR collection bundles without failure, retrieving and regrouping 2317 resources (Patient 88, Encounter 630, MedicationRequest 1597, Organization 2), executed all 20 computable indicators through the compiled-ELM pathway (20/20), and assembled bundles for the 1473-patient synthetic set without per-dataset adaptation. The answer-first manifest specified 1473 synthetic patients across 6569 FHIR entries, covering 35 operationalized definitions spanning 39 indicator codes, with 175 numerator-positive, 434 numerator-negative, 30 denominator-exclusion, 3 boundary, and 95 indicator-specific branches. The 16 medication-overlap indicators contributed 736 control-group patients, including 136 dedicated groups for the early-refill grace-period rule (Table 3); per-group results are reported in Multimedia Appendix 4.

**Table 3:** Summary of synthetic technical and computational verification (answer-first, known-answer design).

Verification component	Scope	Concordant/total	Interpretation
Known-answer indicator results	39 code-derived CQL^a^ libraries	39/39	Internal computational verification
Chronic-prescription grace-period control groups	16 medication-overlap indicators (03-1 to 03-16); positive, negative, and boundary controls	136/136	Expected classifications matched
Independent Python cross-check (differential testing)	39 code-derived libraries, recomputed from generated FHIR^b^ bundles	39/39	Cross-implementation consistency on synthetic fixtures

^a^CQL: Clinical Quality Language.

^b^FHIR: Fast Healthcare Interoperability Resources.

### Synthetic Technical and Computational Verification

The synthetic dataset comprised 1473 patients across FHIR R4 transaction bundles (period May 1 to June 20, 2026) and 4 simulated hospital Organizations; 76.2% (256/336) of patients contributing to the cross-institution fixtures had prescriptions spanning more than one simulated Organization, and the medication-overlap set included 136 dedicated early-refill grace-period groups (Table 3). Multimedia Appendix 4 reports expected outputs for the implemented logic; medication-overlap numerators and denominators are expressed in drug-days, and indicator 13 is reported as mean use rather than a rate.

Across all 39 libraries, platform output matched the author-defined known-answer numerators and denominators (39/39). All 136 chronic-prescription grace-period control groups matched their expected classifications (136/136). As a cross-implementation check, a separately coded Python implementation parsed the generated FHIR bundles and reproduced the CQL/ELM outputs for all 39 libraries (39/39). These fixed-set results establish technical executability and internal computational verification for the implemented logic (Table 2). Table 3 summarizes the 3 verification components.

Testing identified a missing early-refill exemption in indicators 03-1 to 03-16: chronic prescriptions supplied for at least 21 days and refilled within 10 days before prior supply exhaustion should be excluded from overlap [18]. The rule was added, verified in 136 control groups, and applied in both pipelines, demonstrating that testing detected an implementation error before final agreement was obtained.

Full indicator-by-indicator synthetic known-answer results, representative grace-period control-group detail, and the complete 20-indicator real-world differential-testing table are reported in Multimedia Appendix 4.

### Real-World Dataset and FHIR Transformation (Study Population)

The final dataset comprised 88 patients, 65 from Taipei and 23 from Linkou, whose deidentified medication records were electronically exported patient by patient under a predefined specification, with no values manually transcribed. The source long table contained 1597 medication records and 630 visits over January 1 through June 2, 2026. Enrollment was closed at 88 patients. The same records were computed by the platform CQL engine after representation as FHIR R4 Patient, Encounter, and MedicationRequest resources. Representation counts and base FHIR R4 structural validation results are shown in Table 4.

**Table 4:** Real-world Fast Healthcare Interoperability Resources (FHIR) representation counts and base FHIR R4 structural validation (Health Level Seven Application Programming Interface FHIR $validate, specification 4.0.1).

FHIR resource	Source records, n	Computed by CQL^a^ engine (pipeline B), n	Base R4 structural pass, n (%)
Patient	88	88	88 (100)
Encounter	630	630	630 (100)
MedicationRequest	1597	1597	1597 (100)
Organization	2	2	2 (100)
Condition	Not exported	Not applicable	Not applicable
Observation	Not exported	Not applicable	Not applicable
Total	2317	2317	2317 (100)

^a^CQL: Clinical Quality Language.

All 2317 loaded resources passed base FHIR R4 structural validation with no error- or fatal-level issues. Remaining messages were warning- or information-level and did not affect structural validity; the most frequent were the dom-6 narrative best-practice recommendation, information-level notices for the NHI-specific extensions, and warnings that the WHO ATC and NHI medication code systems were not loaded in the local terminology service.

### Real-World Terminology Mapping

NHI 10-digit to WHO ATC mapping coverage was assessed across all 1597 medication records (Table 5). A total of 1570 (98.3%) records mapped to a dedicated ATC code; the 27 unmapped records corresponded to 9 distinct NHI order codes for substances without a dedicated code in the study crosswalk. The dataset contained 410 distinct NHI order codes and 259 distinct ATC codes, with 611 records in the 8 drug classes used by indicators 03-1 to 03-16. All unmapped codes and the 2 NHI codes mapped to more than one ATC code (both S01 ophthalmic combination products) fell outside the studied drug classes and therefore did not change the overlap computations. Because the crosswalk was not compared with an independent expert-adjudicated reference standard, 98.3% is reported as coverage rather than accuracy.

**Table 5:** National Health Insurance (NHI) 10-digit to World Health Organization (WHO) Anatomical Therapeutic Chemical (ATC) terminology-mapping audit (1597 medication records).

Audit item	Result
Total medication records, n	1597
NHI 10-digit → ATC mapped (coverage), n (%)	1570 (98.3)
Unmapped (distinct NHI codes), n	27 (9)
Distinct NHI order codes, n	410
Distinct WHO ATC codes, n	259
Records in the 8 studied drug classes (03-1 to 03-16), n	611
NHI codes mapping to multiple ATC codes (S01 ophthalmic; outside studied classes), n	2

### Initial Aggregate Differential Testing Between 2 Implementations

Table 6 summarizes aggregate concordance and event presence for the 20 computable indicators. Full indicator-level numerators and denominators are provided in Multimedia Appendix 4.

The Python reference implementation and the platform CQL engine produced identical aggregate numerators and denominators for all 20 computable indicators (20/20), with no discordant indicator-level total observed. The observed outpatient injection, outpatient antibiotic, chronic continuous-prescription, and ≥10-drug-item rates were 2.5% (16/630), 4.1% (26/630), 54.3% (342/630), and 0% (0/630), respectively. Same-institution medication-overlap rates ranged from 1.4% to 9.3%. Overall, 7 of the 8 cross-institution drug-class series had nonzero overlap, with rates among those series ranging from 0.1% to 2.4%.

**Table 6:** Summary of real-world differential testing by indicator group (n=88).

Indicator group	Codes	Indicators, n	Aggregate N/D^a^ concordance	Nonzero numerator
Outpatient medication use and prescribing	01, 02, 04, 05	4	4/4	3/4
Same-institution medication overlap	03-1 to 03-8	8	8/8	8/8
Cross-institution medication overlap	03-9 to 03-16	8	8/8	7/8
Total	01-05 and 03-1 to 03-16	20	20/20	18/20

^a^N/D: numerator/denominator.

### Execution Traceability Output

Indicator 03-11 (cross-institution antidiabetic overlap; NHI code 1715) illustrates the execution trace. Its observed value of 1.9% (33/1658 drug-days) was associated with CQL library version 1.0.0, the query period 2026-01-01 to 2026-06-02, drug class WHO ATC A10, institutional boundaries defined by distinct source identifiers, and the grace-period rule. The denominator resolved to 9 patients and 71 A10 MedicationRequest resources and the numerator to 3 patients, 7 MedicationRequest resources and 4 overlapping pairs, each contributing drug-day traceable to its MedicationRequest, dispense validity period and retained institutional identifier. Formal audit reports, FHIR AuditEvent logging and external provenance verification remain future work.

### Platform Performance

All 20 real-world indicators were executed through the compiled-ELM pathway in the local self-hosted HAPI FHIR research environment. At the deployed HTTP service, end-to-end response time had a median of 13526 ms and a 95th percentile of 24730 ms across the 20 indicator calls (minimum 4676 ms, maximum 28110 ms). In the engine-module instrumentation, FHIR retrieval accounted for 54.6% of summed elapsed time, per-patient bundle assembly for 1.3%, and ELM evaluation for 44%. The implementation requeries resources during full indicator-set execution, so single-pass retrieval with caching is the primary optimization target. Stage-resolved and per-indicator timings are reported in Multimedia Appendix 2. These measurements come from a single unoptimized environment and are not a general performance benchmark.

## Discussion

### Principal Findings

#### Technical and Computational Findings

We developed 39 executable CQL libraries derived from Taiwan NHI medical-center quality indicator codes and evaluated their execution using synthetic and real-world data. All 39 libraries compiled to ELM and produced the expected results in the predefined synthetic fixtures, including all 136 early-refill grace-period control groups. In the 88-patient real-world cohort, the Python reference implementation and the CQL pipeline produced identical aggregate numerators and denominators for all 20 computable indicators. All 2317 FHIR resources passed base FHIR R4 structural validation, and ATC mapping was available for 98.3% (1570/1597) of medication records. These findings support technical executability and computational consistency of the implemented logic. Further evaluation is required for semantic fidelity, patient-level agreement, and portability across systems.

#### The Library as an Executable Specification

The 39 libraries were authored deliberately, including those that depend on laboratory results represented as LOINC-coded Observation resources, so that the set identifies resource requirements beyond the current export. Only 20 indicators could be evaluated on real data because Condition, Observation, and specific encounter-type data were unavailable (Table 1; Multimedia Appendix 3). Synthetic testing of the remaining libraries confirms execution against predefined outputs, not fidelity to complete official specifications. What the library does establish is a specification roadmap and a concrete target for future data-export upgrades: the scope reductions in Table 1 represent a requirements list rather than a shortfall.

### Comparison With Prior Work

Prior work established that representing measure and phenotype logic in CQL can enable portable, cross-platform execution, though a scoping review documented rapid proliferation alongside limited evidence of validated production deployment [20]. Brandt et al [21] translated a heart-failure phenotype into CQL and obtained identical cohorts across an Observational Medical Outcomes Partnership instance and a FHIR-based platform, and in a later 3-site study of a thrombotic-event phenotype reported 100% precision and recall at 2 sites and 95% precision and 84% recall at the third [22]. These studies provided chart-reviewed validation of individual phenotypes. Our study evaluated a larger set of national quality indicator codes, although validation was limited to aggregate differential testing rather than chart-level adjudication. Related frameworks have similarly emphasized reusable, portable logic over site-specific queries [23,24].

CQL infrastructure studies have examined embedded-engine performance [5], LLM-assisted execution [6], distributed feasibility queries [25], and national surveillance architectures [26]. We focused on practical deployment issues and on linking computed indicator results to source records, CQL versions, and query parameters.

von Dincklage et al [9] emphasized clinical-semantic validation through iterative expert review but did not evaluate execution on real clinical systems. Our study instead included real-world execution, although formal expert adjudication remains pending. Medical Information Mart for Intensive Care IV on FHIR demonstrated FHIR transformation and validation at substantially greater data volume [27], whereas this study focused on executable quality-indicator logic in a smaller dataset.

Terminology mapping will become more complex when the library is extended beyond medication-related indicators. In this study, the source data already used standardized NHI codes. Hospitals starting from local code sets would require an additional mapping step before FHIR resources could be generated, such as mapping local medication codes to NHI codes and WHO ATC, laboratory codes to LOINC, and diagnosis codes to ICD-10-CM. This local-to-standard mapping process was not evaluated in this study and may introduce institution-specific errors.

Ambiguity can also remain after standardization. A single NHI medication code may correspond to more than one ATC concept, particularly for combination products, as observed for 2 S01 ophthalmic codes in our cohort. A terminology server can provide curated and version-controlled mappings [28], but ambiguous mappings may still require predefined rules or expert review. Several strategies can reduce these conflicts. Comparison at an aggregated ATC level absorbs many combination-product ambiguities, and dedicated WHO ATC combination codes can be used where they exist. Codes that still map to more than one concept can be narrowed by an indicator-scoped whitelist or referred for expert adjudication under a documented primary-mapping rule. Such decisions should be documented together with the indicator logic to support reproducibility.

Our cross-institution medication-overlap indicators address the same clinical concern as Taiwan’s PharmaCloud clinical decision support evaluated by Yang et al [29], whose 3-month operational study covered 178,300 outpatient prescriptions and found potential duplicate medication in 5.8% of eligible prescriptions, with physicians canceling 42.1% of alerted orders. In our dataset, 7 of 8 cross-institution drug-class series showed nonzero overlap, at 0.1% to 2.4% of drug-days. Because Yang et al [29] reported prescription-level alerts whereas our indicators were calculated using drug-days within predefined drug classes, the percentages are not directly comparable. Their study demonstrates the clinical use of PharmaCloud at scale [29], while our analysis evaluates whether cross-institution medication-overlap logic can be represented and executed using CQL and FHIR.

### Clinical and Policy Implications

The platform supports review of cross-institution medication overlap by linking indicator results to source records, rule versions, and query parameters. Such review is relevant to polypharmacy and medication reconciliation [30,31]. Reusable CQL definitions may also reduce repeated implementation work in quality reporting [7,32]. These potential benefits require evaluation: the study did not measure workflow impact, prescribing changes, or clinical outcomes.

Taiwan’s national medication query service supplies cross-institution records that may be unavailable in other settings. Point-of-care delivery through clinical decision support (CDS) Hooks [33] remains future work; this output is for research use.

### Limitations

This study has several limitations. The study establishes execution and computational agreement, not complete fidelity to official NHI measures. Author-defined fixtures lacked independent clinical or claims-expert adjudication. Some denominators, exclusions, code lists, and temporal rules were adapted or incomplete, including visit-based denominators, broader ATC scopes, and surgical terminology proxies (Multimedia Appendix 3). These differences preclude interpreting outputs as validated official NHI rates.

The convenience sample included only 88 patients from 2 campuses of one health system, limiting representativeness and assessment of rare events. Patient-by-patient export was labor-intensive and unsuitable for scaling. External institutions supplied records through the cloud service but were not participating study sites.

The medication-only export and bounded look-back window fragment the longitudinal record, so the indicators describe a partial rather than a holistic view of a patient’s care, omitting inpatient, diagnosis, and laboratory data. Records were retrieved through the national cloud service rather than exchanged directly, with duplicates removed where hospital and cloud records overlapped. Missing prescriptions or incomplete treatment histories can affect overlap estimates; the available data do not establish the direction or magnitude of that effect. This fragmentation matters for medication-safety monitoring, which depends on a record that is complete across prescribers and care settings. Real-world findings therefore apply only to the supported outpatient medication indicators.

External portability remains undemonstrated. All indicators were executed on synthetic and real-patient FHIR bundles in a self-hosted local HAPI FHIR environment, but the libraries were not run against patient-level data on an external national or institutional server; the public THAS/metadata check tested connectivity only, not resource retrieval, terminology compatibility, or CQL execution.

The differential-testing design shared its source data. Both pipelines began with the same electronic export and implemented the same operationalized definitions, even though they used different representations and separately coded computations. Agreement therefore evaluates transformation and implementation consistency rather than independent clinical adjudication. Comparison was performed at the aggregate numerator-denominator level, so offsetting patient-level or event-level discrepancies cannot be excluded. The design also cannot detect an error in the source export or a rule misinterpretation shared by both pipelines.

Local-to-standard terminology mapping was not tested because source records already used standardized NHI codes. Institutions using local codes would need additional mapping and validation before adopting the libraries.

### Future Directions

Further evaluation should begin with independent review by clinical, claims, and NHI experts of rule semantics against official requirements, followed by patient- and event-level comparisons with adjudication of discordant cases and ambiguous terminology mappings.

Portability testing should assess TW Core and project-specific profiles, versioned terminology mappings, a second CQL engine, and independent institutions, including local-to-standard code mapping. Performance benchmarks should specify hardware, request counts, sample sizes, caching, and concurrency.

Subsequent multicenter studies should use larger longitudinal datasets with inpatient, procedure, and outcome records, explicit sampling, and adequate rare-event representation. Bulk export and patient-level completeness reporting would support this work. Clinical studies would then evaluate delivery through CDS Hooks and its effect on care.

### Conclusions

This study demonstrates executable, source-traceable CQL implementations derived from Taiwan NHI quality indicators, with synthetic verification and aggregate agreement between independently coded pipelines for 20 real-world indicators. The library is not a validated replacement for official NHI computation. Independent semantic adjudication and patient-level, cross-system, and multicenter evaluations are needed before broader use.

## Data Availability

Selected Clinical Quality Language/Expression Logical Model implementation materials and platform source code are available in a public development repository [34], with a separate live demonstration [35]. The demonstration contains no research patient data and uses Taiwan Health Application Space only for a public/metadata CapabilityStatement connectivity check. The repository is under active development and does not yet provide a version-tagged research release with a fixed license, dependency lock information, or packaged reproducibility instructions. The deidentified National Health Insurance (NHI) outpatient claims and cross-institution medication records analyzed for real-world differential testing cannot be shared. Under NHI data-governance rules and the terms of the institutional review board approval, the authors have no authority to grant third-party access to these records, and there is therefore no application route to the authors.

## Appendix

Multimedia Appendix 1 Completed STARE-HI reporting checklist.

Multimedia Appendix 2 Clinical Quality Language/Expression Logical Model execution evidence and response-time measurements.

Multimedia Appendix 3 Indicator specifications, terminology mappings, Fast Healthcare Interoperability Resources field footprint, and comparison with official National Health Insurance definitions.

Multimedia Appendix 4 Synthetic and real-world verification results.

## Abbreviations

ATC: Anatomical Therapeutic Chemical
CDS: clinical decision support
CORS: cross-origin resource sharing
CQL: Clinical Quality Language
DDD: defined daily dose
ELM: Expression Logical Model
ETL: extract-transform-load
FHIR: Fast Healthcare Interoperability Resources
HAPI: Health Level Seven Application Programming Interface
HIPAA: Health Insurance Portability and Accountability Act
HL7: Health Level Seven
ICD: International Statistical Classification of Diseases
ICD-10-CM: International Classification of Diseases, Tenth Revision, Clinical Modification
LLM: large language model
LOINC: Logical Observation Identifiers Names and Codes
NHI: National Health Insurance
SMART: Substitutable Medical Applications, Reusable Technologies
SNOMED CT: Systematized Nomenclature of Medicine Clinical Terms
STARE-HI: Statement on Reporting of Evaluation Studies in Health Informatics
THAS: Taiwan Health Application Space
TW Core: Taiwan Core
WHO: World Health Organization
