# Supplementary material for: Implementing Taiwan National Health Insurance Quality Indicators as Clinical Quality Language on Fast Healthcare Interoperability Resources: Development and Feasibility Study
Source: JMIR Med Inform. 2026 Oct 15;14:e108093. doi: 10.2196/108093 (PMC13720404; doi:10.2196/108093)
Supplement: Supplementary file 1 [file medinform_v14i1e108093_app1.docx]

**Multimedia Appendix 1**

**STARE-HI Reporting Checklist**

*Statement on the Reporting of Evaluation Studies in Health Informatics — official item list per Talmon et al. (2009), Table 1.*

**Manuscript:** Implementing Taiwan National Health Insurance Quality Indicators as Clinical Quality Language on FHIR: Development and Feasibility Study (JMIR Medical Informatics, #108093)

*All 35 items and their numbering are reproduced exactly from the official STARE-HI statement. Item locations refer to manuscript sections and subsections. Where an official item is not applicable to this retrospective, deidentified development-and-feasibility study, this is stated within that item's row (e.g., item 7.2).*

| **#** | **STARE-HI item (official)** | **How and where addressed in the manuscript** | **Location (section)** |
| --- | --- | --- | --- |
| 1 | **Title** | Title identifies the evaluated system (a FHIR/CQL platform for NHI quality indicators) and the study type (development and feasibility study). | Title |
| 2 | **Abstract** | Structured abstract: Background, Objective, Methods, Results, Conclusions. | Abstract |
| 3 | **Keywords** | FHIR; Clinical Quality Language; electronic clinical quality measures; reproducibility; data provenance; medication safety; cross-institution; National Health Insurance. | Keywords |
| **4** | **Introduction** | Provides background and rationale (see 4.1–4.3). | Introduction |
| 4.1 | **Scientific background** | Quality measurement moving to machine-executable specifications; FHIR + CQL; systematic-review gaps in validated deployment [1–4]. | Introduction (paras 1–2) |
| 4.2 | **Rationale for the study** | Portability tension; credibility boundary of automated computation; Taiwan single-payer setting; NHI indicators not previously implemented as executable CQL. | Introduction (paras 3–5) |
| 4.3 | **Objectives of study** | Study objective and four contributions stated; ethics/IRB approval (No. 202601223B0) and consent waiver reported. | Abstract – Objective; Introduction (para 6); Ethical Considerations |
| **5** | **Study context** | Setting and system described (see 5.1–5.2). | Methods – Study Design; Platform Architecture |
| 5.1 | **Organizational setting** | Taipei and Linkou Chang Gung Memorial Hospitals; local self-hosted FHIR R4 research environment. | Methods – Study Design and Overview; Results – Real-World Dataset |
| 5.2 | **System details and system in use** | FHIR R4-native platform; architecture in the main text with the full component-version table in Multimedia Appendix 2; 39 CQL libraries; 6 FHIR resources / 21 data elements; cross-institution overlap algorithm; 3 deployment-layer findings. | Methods – Platform Architecture; CQL Indicator Library; FHIR Data Model; Cross-Institution Medication Overlap Algorithm; Implementation Notes; Multimedia Appendix 2 |
| **6** | **Methods** | Study design and methods (see 6.1–6.7). | Methods |
| 6.1 | **Study design** | Two-part design (synthetic technical/computational verification; retrospective 2-campus aggregate differential testing); 3-level evidence framework. | Methods – Study Design and Overview; Evaluation Framework; Evaluation 1; Evaluation 2; Tables 1–2 |
| 6.2 | **Theoretical background** | Conceptual basis is the FHIR/CQL/ELM standards model and a predefined 3-level evidence framework; no behavioral or user-acceptance theory applies to this technical evaluation. | Methods – Evaluation Framework; Table 2 |
| 6.3 | **Participants** | No participants were prospectively recruited or contacted; the analysis used deidentified records from 88 patients. The cohort is described as a convenience sample, with the reason for pragmatic selection, the absence of a prespecified sampling frame, the absence of inclusion/exclusion criteria beyond record availability, and the absence of post-selection exclusions all stated. Cohort scope, the reason for each reduction, and record export are described; consent was waived. | Methods – Evaluation 2; Table 1; Ethical Considerations |
| 6.4 | **Study flow** | Study periods and dates (real-world Jan 1–Jun 2 2026; synthetic simulated May 1–Jun 20 2026); data flow from claims through FHIR transformation, CQL execution, and reporting. | Methods – Evaluation 1; Evaluation 2; Figure 1; Results – Real-World Dataset |
| 6.5 | **Outcome measures or evaluation criteria** | Numerator, denominator, and rate; aggregate concordance (exact equality); base FHIR R4 structural pass rate; NHI→ATC mapping coverage; execution time. | Methods – Evaluation Framework; Analysis; Table 2 |
| 6.6 | **Methods for data acquisition and measurement** | Deidentified NHI outpatient claims + national cloud cross-institution medication records; patient-by-patient electronic export to a long table; FHIR transformation; execution-time measurement at two levels. | Methods – Study Design; Evaluation 2 (Stages A–D); Execution-Time Measurement; Ethical Considerations |
| 6.7 | **Methods for data analysis** | Two separately coded pipelines (Python reference; platform CQL engine); aggregate concordance defined as exact numerator/denominator equality; descriptive statistics; rationale for reporting no confidence interval. | Methods – Analysis; Evaluation 2 (Stage D) |
| **7** | **Results** | Findings reported (see 7.1–7.4). | Results |
| 7.1 | **Demographic and other study coverage data** | Study size: 88 patients (Taipei 65 / Linkou 23), 1597 medication records, 630 visits, 2317 FHIR resources; synthetic set of 1473 patients / 136 control groups. Patient-level demographics are not reported, as the deidentified dataset is a methodological substrate, not an epidemiological sample. | Results – Real-World Dataset and FHIR Transformation; Evaluation 1 |
| 7.2 | **Unexpected events during the study** | *Not applicable — no external events (system outages, protocol deviations, staff changes) affected the retrospective, deidentified dataset.* | — |
| 7.3 | **Study findings and outcome data** | 39/39 libraries passed synthetic verification; 136/136 grace-period controls; 20/20 aggregate concordance; 98.3% ATC coverage; all 2317 resources passed structural validation; per-indicator results and execution times. | Results (full section); Multimedia Appendices 2 and 4 |
| 7.4 | **Unexpected observations** | A missing early-refill grace-period rule was discovered during development, implemented, and verified against 136 control groups; a reproducible UTF-8 BOM silent compilation failure was documented. | Methods – Implementation Notes; Results (grace-period description) |
| **8** | **Discussion** | Interpretation and assessment (see 8.1–8.5). | Discussion |
| 8.1 | **Answers to study questions** | Principal Findings: NHI indicator logic can be expressed as executable CQL and computed reproducibly; computational agreement is not clinical adjudication. | Discussion – Principal Findings |
| 8.2 | **Strengths and weaknesses of the study** | Six limitations (evidence levels interpreted separately; small single-system cohort; cloud-data constraints; external portability undemonstrated; shared-source differential design; incomplete fidelity to official definitions); strengths noted in Principal Findings. | Discussion – Principal Findings; Limitations |
| 8.3 | **Results in relation to other studies** | Comparison with Brandt, Kiel, Eapen, von Dincklage, and Yang et al., stating what is novel. | Discussion – Comparison With Prior Work |
| 8.4 | **Meaning and generalisability of the study** | Clinical and policy implications (all stated as conditional); generalisability caveats. | Discussion – Clinical and Policy Implications; Limitations |
| 8.5 | **Unanswered and new questions** | Future Directions: three phases (semantic validation; interoperability validation; clinical implementation). | Discussion – Future Directions |
| 9 | **Conclusion** | Summarizes findings; states this is a documented foundation, not a validated replacement for official NHI computation; outlines phased validation. | Conclusions |
| 10 | **Authors' contribution** | Author contributions stated. | Authors' Contributions |
| 11 | **Competing interests** | No financial competing interests declared. As STARE-HI item 11 requires, the manuscript explicitly states the relation of the study team to the system evaluated: the authors are the developers of the platform and CQL indicator library, so the evaluation was performed by the development team rather than by an independent external group. | Conflicts of Interest |
| 12 | **Acknowledgement** | Financial support is acknowledged in the Funding section (NSTC grant 115-2314-B-182A-050-MY2). Generative AI use is disclosed under its own separate subheading, as required by JMIR policy; the submitted version's Acknowledgments section contained only that statement, so it is now titled Generative AI Disclosure and there is no separate Acknowledgments section. | Funding; Generative AI Disclosure |
| 13 | **References** | Complete reference list per journal style. | References |
| 14 | **Appendices** | Multimedia Appendix 1 (this checklist); Appendix 2 (execution evidence); Appendix 3 (indicator specifications, mappings, adaptations); Appendix 4 (verification results). Selected code/materials in a public repository. | Multimedia Appendices 2–4; Data Availability |

References

1. Talmon J, Ammenwerth E, Brender J, de Keizer N, Nykänen P, Rigby M. STARE-HI—Statement on reporting of evaluation studies in Health Informatics. Int J Med Inform. 2009;78(1):1-9. doi:10.1016/j.ijmedinf.2008.09.002

2. Brender J, Talmon J, de Keizer N, Nykänen P, Rigby M, Ammenwerth E. STARE-HI—Statement on Reporting of Evaluation Studies in Health Informatics: explanation and elaboration. Appl Clin Inform. 2013;4(3):331-358. doi:10.4338/ACI-2013-04-RA-0024
