# Supplementary material for: Implementing Taiwan National Health Insurance Quality Indicators as Clinical Quality Language on Fast Healthcare Interoperability Resources: Development and Feasibility Study
Source: JMIR Med Inform. 2026 Oct 15;14:e108093. doi: 10.2196/108093 (PMC13720404; doi:10.2196/108093)
Supplement: Supplementary file 2 [file medinform_v14i1e108093_app2.docx]

**Multimedia Appendix 2**

**CQL/ELM Execution Evidence and Response-Time Measurements**

**Associated manuscript: Implementing Taiwan National Health Insurance Quality Indicators as Clinical Quality Language on FHIR: Development and Feasibility Study**

# Execution Environment

| **Component** | **Version or configuration** |
| --- | --- |
| FHIR specification | FHIR R4 4.0.1 |
| FHIR server | Self-hosted HAPI FHIR Server 8.10.0 (local research environment) |
| CQL compiler | cql-to-elm 3.10.0 |
| CQL execution | cql-execution 2.4.6 |
| FHIR data adapter | cql-exec-fhir 2.1.6 (PatientSource.FHIRv401()) |
| Server runtime | Node.js 22.20.0; Express 4.22.1 |
| Visualization | Chart.js 4.4.0 |
| Research execution path | Local HAPI FHIR retrieval → patient-centric resource assembly → compiled-ELM execution |
| Public demonstration | THAS /metadata CapabilityStatement connectivity check only; no research data or indicator execution |

# Execution-Path Manifest

The manifest below identifies the execution provenance for the real-world compiled-ELM run.

| **Manifest item** | **Value** |
| --- | --- |
| CQL compiler / execution / FHIR adapter | cql-to-elm 3.10.0 / cql-execution 2.4.6 / cql-exec-fhir 2.1.6 |
| FHIR specification | 4.0.1 |
| Measurement period | 2026-01-01 to 2026-06-02 |
| Execution date | 2026-07-27 |
| Patient bundles executed | 88 (Patient, Encounter, MedicationRequest; Organization as supporting reference) |
| Indicators executed | 20 (01, 02, 04, 05, and 03-1 to 03-16) |
| Resources loaded and validated | 2317 (Patient 88, Encounter 630, MedicationRequest 1597, Organization 2) |

# Real-World compiled-ELM Execution and Response Times

All 20 computable indicators executed successfully through the compiled-ELM pathway on the final 88-patient cohort (1597 medication records, 630 visits). Numerators and denominators were identical to the independent Python reference implementation (pipeline A; 20/20 concordant). End-to-end API response time was measured from FHIR resource retrieval through per-patient bundle assembly to ELM execution.

**Table A3-1. Per-indicator compiled-ELM results and end-to-end API response time (n=88).**

| **Code** | **D** | **N** | **Rate %** | **Unit** | **API (ms)** |
| --- | --- | --- | --- | --- | --- |
| 01 | 630 | 16 | 2.54 | visits | 15018 |
| 02 | 630 | 26 | 4.13 | visits | 9132 |
| 03-1 | 5176 | 479 | 9.25 | drug-days | 14154 |
| 03-2 | 3111 | 97 | 3.12 | drug-days | 8154 |
| 03-3 | 1658 | 130 | 7.84 | drug-days | 7345 |
| 03-4 | 1020 | 18 | 1.76 | drug-days | 12244 |
| 03-5 | 502 | 7 | 1.39 | drug-days | 22029 |
| 03-6 | 1649 | 33 | 2.00 | drug-days | 9737 |
| 03-7 | 1148 | 43 | 3.75 | drug-days | 4676 |
| 03-8 | 584 | 14 | 2.40 | drug-days | 10825 |
| 03-9 | 5176 | 50 | 0.97 | drug-days | 12897 |
| 03-10 | 3111 | 8 | 0.26 | drug-days | 24689 |
| 03-11 | 1658 | 33 | 1.99 | drug-days | 11162 |
| 03-12 | 1020 | 1 | 0.10 | drug-days | 28110 |
| 03-13 | 502 | 1 | 0.20 | drug-days | 17393 |
| 03-14 | 1649 | 3 | 0.18 | drug-days | 16040 |
| 03-15 | 1148 | 27 | 2.35 | drug-days | 5538 |
| 03-16 | 584 | 0 | 0.00 | drug-days | 23147 |
| 04 | 630 | 342 | 54.29 | visits | 16811 |
| 05 | 630 | 0 | 0.00 | visits | 24730 |

Across the 20 indicators, the end-to-end API response time had a median of 13526 ms, a 95th percentile of 24730 ms, a minimum of 4676 ms, and a maximum of 28110 ms; no execution failures occurred. For the overlap indicators (03-1 to 03-16) the numerator and denominator are in drug-days; for indicators 01, 02, 04, and 05 the unit is visits, reflecting the denominator deviation documented in Multimedia Appendix 3.

The reported median is the standard median (mean of the two middle values) of the 20 per-indicator response times.

# Stage-Resolved Timing Decomposition

To locate where end-to-end time is spent, the execution pipeline was separately instrumented with process.hrtime.bigint() at the engine-module level, dividing each indicator run into 3 consecutive stages: (1) FHIR resource retrieval, comprising paged queries to the local HAPI FHIR R4 server; (2) per-patient bundle assembly, in which resources are regrouped into FHIR collection bundles and loaded through cql-exec-fhir PatientSource.FHIRv401(); and (3) ELM evaluation by cql-execution. All 20 indicators were forced through the compiled-ELM pathway, with no JavaScript shortcut computation, so stage 3 represents the true cost of evaluating compiled ELM. Six rounds were run against the 88-patient cohort; the first round was discarded as cold start (JIT warm-up and connection establishment), leaving 5 rounds and 100 stage-resolved samples.

**Table A3-2.** Stage-resolved decomposition across all 20 indicators (5 rounds, 100 samples).

| **Stage** | **Median (ms)** | **P95 (ms)** | **Share of total elapsed time** |
| --- | --- | --- | --- |
| FHIR resource retrieval | 5263 | 5563 | 54.6% |
| Per-patient bundle assembly | 110 | 205 | 1.3% |
| ELM evaluation (cql-execution) | 2757 | 10908 | 44.0% |
| Instrumented end-to-end total | 7866 | 16275 | 100.0% |

**Table A3-3.** Per-indicator ELM evaluation and bundle assembly times.

| **Indicator** | **ELM evaluation median (ms)** | **ELM evaluation P95 (ms)** | **Bundle assembly median (ms)** |
| --- | --- | --- | --- |
| 01 | 10974 | 12735 | 104 |
| 02 | 9660 | 12037 | 108 |
| 03-1 | 5586 | 5852 | 101 |
| 03-2 | 3032 | 3262 | 114 |
| 03-3 | 3391 | 3518 | 122 |
| 03-4 | 1853 | 2140 | 119 |
| 03-5 | 1715 | 1911 | 95.5 |
| 03-6 | 2247 | 2467 | 106 |
| 03-7 | 1974 | 2416 | 106 |
| 03-8 | 1785 | 3017 | 103 |
| 03-9 | 5676 | 6228 | 117 |
| 03-10 | 2901 | 2978 | 118 |
| 03-11 | 3352 | 3728 | 94.9 |
| 03-12 | 1800 | 2307 | 102 |
| 03-13 | 1828 | 1929 | 104 |
| 03-14 | 2490 | 3812 | 110 |
| 03-15 | 2268 | 3011 | 129 |
| 03-16 | 2133 | 4567 | 116 |
| 04 | 2724 | 4271 | 133 |
| 05 | 9671 | 13834 | 110 |

Stage shares were computed by summing elapsed time per stage across all samples and dividing by the summed total, a quantity that is additive. Stage medians are reported separately and are not additive: the 3 stage medians do not sum to the instrumented end-to-end median.

The instrumented end-to-end median of 7866 ms differs from the 13526 ms reported in Table A3-1 because the two measurements are taken at different levels: Table A3-1 measures the deployed HTTP service through the full Express stack, whereas the decomposition calls the engine modules directly. The result that transfers across environments is the relative distribution across stages, not the absolute millisecond values.

FHIR resource retrieval is the dominant cost. Its share is conservative: in the deployed service each indicator independently re-queries the FHIR server, so running the full set retrieves the same patient resources 20 times, whereas the decomposition retrieved once per round and attributed that cost across indicators, matching the end-to-end behavior of a single-indicator request. Single-pass retrieval with caching is therefore the primary optimization target, and it does not involve the CQL engine.

# Requirements for a Future Reproducible Benchmark

The measurements above characterize the current deployment but are not a benchmark: workload, environment, and concurrency were not prespecified, and all runs used a single unoptimized research environment. The stage-resolved decomposition satisfies the repetition and stage-attribution requirements below; the remaining items are still required for a fully reproducible benchmark.

| **Reporting item** | **Required report** |
| --- | --- |
| Hardware and operating system | Processor, memory, storage, operating system |
| Dataset and payload | Patients, FHIR resources, bundle size, indicators executed |
| Warm-up and cache state | Warm-up calls and cold/warm-cache definition |
| Repetitions and concurrency | Number of repetitions and concurrent requests |
| Timing boundary | FHIR retrieval included/excluded; start and stop events |
| Primary results | Median, interquartile range, 95th percentile, minimum, maximum |
| Failure reporting | Timeouts, failed calls, validation errors |

# Finding 1: Deliberate Reproduction of the Byte-Order Mark Compilation Failure

The compilation failure attributed to a UTF-8 byte-order mark (BOM) was reproduced deliberately so that the mechanism rests on a repeatable demonstration rather than on build history. The project’s own Gradle build task was used rather than an ad hoc script, so that the reproduction reflects the production build path. The input directory contained the library under test together with its unmodified dependency FHIRHelpers-4.0.1.cql, isolating the BOM on the library under test as the only variable.

**Table A3-4.** Reproduction environment and build invocation.

| **Item** | **Value** |
| --- | --- |
| CQL library under test | Indicator_01_Outpatient_Injection_Usage_Rate_3127.cql |
| CQL translator | cql-to-elm 3.10.0 (info.cqframework:cql-to-elm-cli) |
| Java runtime | OpenJDK 17.0.17 (Microsoft build 17.0.17+10-LTS) |
| Operating system | Microsoft Windows 11, build 26200, 64-bit |
| Build tool | Gradle 8.11, cql2elm JavaExec task |
| Invocation | org.cqframework.cql.cql2elm.cli.Main --input <dir> --output <dir> --format JSON --signatures Overloads --disable-list-demotion --disable-list-promotion |

**Table A3-5.** Three-step reproduction: baseline, BOM injection, and restoration.

| **Step** | **First 3 bytes (hex)** | **Input size (bytes)** | **Translation** | **ELM output** |
| --- | --- | --- | --- | --- |
| 1. Baseline, no BOM | 6c 69 62 ("lib") | 12119 | Succeeded | 260203 bytes |
| 2. BOM injected | ef bb bf (BOM) | 12122 | Failed | No file produced |
| 3. BOM removed | 6c 69 62 ("lib") | 12119 | Succeeded | 260203 bytes |

The translator reported, verbatim: "Translation failed due to errors: Error:[1:0, 1:0] Syntax error". No ELM file was produced for the library under test; the output directory contained only the ELM for the co-located dependency FHIRHelpers-4.0.1.cql (623855 bytes), which carried no BOM and translated successfully in the same batch. Removing the BOM restored compilation with byte-identical ELM output (260203 bytes), which excludes causes other than the BOM.

The failure is silent at the build level. The translator exits with status 0 when an individual file fails and skips that file, so Gradle reported BUILD SUCCESSFUL; the fault was observable only as the absence of one expected ELM file. This is the reason the original occurrence was initially misread as a CQL syntax error, and it is the practical hazard the pre-compilation BOM check is intended to remove.

Evidence files retained: baseline_compile.log, bom_compile.log (containing the verbatim error), and restored_compile.log; the three input states of the CQL file; and the corresponding ELM output directories, in which the BOM output directory lacks the indicator file.

# References

1. HL7 International. FHIR Release 4 (R4) specification. URL: https://hl7.org/fhir/R4/ [accessed 2026-07-22]

2. HL7 International. Clinical Quality Language (CQL) specification, release 1, STU 5. URL: https://cql.hl7.org/ [accessed 2026-07-22]

3. HAPI FHIR. HAPI FHIR documentation. URL: https://hapifhir.io/hapi-fhir/docs/ [accessed 2026-07-22]
