# Supplementary material for: Implementing Taiwan National Health Insurance Quality Indicators as Clinical Quality Language on Fast Healthcare Interoperability Resources: Development and Feasibility Study
Source: JMIR Med Inform. 2026 Oct 15;14:e108093. doi: 10.2196/108093 (PMC13720404; doi:10.2196/108093)
Supplement: Supplementary file 3 [file medinform_v14i1e108093_app3.docx]

**Multimedia Appendix 3**

**Indicator Specifications, Terminology Mappings, FHIR Field Footprint, and Comparison With Official NHI Definitions**

**Associated manuscript: Implementing Taiwan National Health Insurance Quality Indicators as Clinical Quality Language on FHIR: Development and Feasibility Study**

# Indicator Categories and Principal Terminology

The 39 Taiwan National Health Insurance (NHI) medical-center quality indicators are grouped into 5 categories. The real-world medication and outpatient subset primarily uses NHI order codes and World Health Organization Anatomical Therapeutic Chemical codes; some surgical indicators use synthetic-only terminology approximations.

| **Category** | **Indicators, n** | **Official NHI code series** | **Principal terminology** |
| --- | --- | --- | --- |
| Medication safety | 19 | 01, 02, 03-1 to 03-16, 05 | WHO ATC; NHI drug & order codes |
| Outpatient quality | 4 | 04, 06, 07, 08 | NHI order codes; WHO ATC |
| Inpatient quality | 2 | 09, 10 | NHI codes; ICD-10-CM |
| Surgical quality | 11 | 11-1 to 11-4, 12, 13, 14, 15-1 to 15-3, 19 | NHI procedure codes; ICD-10-CM |
| Outcome quality | 3 | 16, 17, 18 | ICD-10-CM; LOINC |

# Implementation Maturity

| **Maturity** | **Indicators, n** | **Description** |
| --- | --- | --- |
| Implementation-complete (5/5) | 39 | Functional level ≥4; quarterly statistics, business helpers, exclusion rules, numerator/denominator/rate |
| Lacking quarterly statistics | 0 | None; all 39 prototype libraries reached implementation completeness |
| Total | 39 |  |

Note: Maturity refers to build completeness of the prototype CQL libraries (functional level, period aggregation, business-rule helpers, exclusion logic, and numerator/denominator/rate outputs). It does not indicate fidelity to the complete official NHI technical definitions, which varies by indicator and is reported separately in Table A1-4.

# FHIR R4 Field Footprint

The platform uses 6 principal clinical FHIR R4 resource types—Patient, Encounter, MedicationRequest, Observation, Condition, and Procedure—with Organization as a supporting institutional reference. Across all 39 indicators, 21 of 190 standard data elements are activated. The institutional boundary for cross-institution computation is the source NHI medical-institution identifier retained with each record. The real-world bundle instantiated 2 Organization resources for the 2 participating campuses; identifiers from other institutions were retained as source provenance rather than instantiated as separate Organization resources.

| **FHIR R4 resource** | **Role in the platform** | **Real-world medication/outpatient subset** |
| --- | --- | --- |
| Patient | Patient identity and linkage context | Used |
| Encounter | Visit context and institutional boundary | Used |
| MedicationRequest | Medication, days supplied, and prescribing institution | Used |
| Observation | Laboratory results | Planned for indicators 06-08 |
| Condition | Diagnosis context | Planned for indicators 06-08 |
| Procedure | Surgical/procedural events | Synthetic verification only |
| Organization | Supporting reference for medical-institution provenance | Used |

# Comparison With Official NHI Technical Definitions

Table A1-4 paraphrases the official 2023 NHI professional-edition definitions and compares them with the current CQL implementation or synthetic-data study design. English translations were prepared by the authors. Page numbers refer to the cited official technical report.

| **Code** | **Indicator** | **Official numerator/denominator and key business rules** | **Current CQL/study status** | **Known deviation or implementation limitation** |
| --- | --- | --- | --- | --- |
| 01 | Outpatient injection use rate (3127) | Numerator: medication-administration claims for injectable drugs (10-digit NHI order code with eighth digit=2). Denominator: all medication-administration claims. Seven numerator exclusions are specified: self-injection drugs, outpatient chemotherapy, emergency injections, influenza vaccine, tetanus toxoid, outpatient surgery drugs, prior-authorization drugs, and STAT drugs (p 2). | Evaluated by aggregate differential testing on real-world data. The current implementation uses visits as the denominator rather than medication-administration claims. | Denominator granularity differs (visit vs medication-administration claim); completeness of all 7 exclusions was not evaluated in this study. |
| 02 | Outpatient antibiotic use rate (1140.01) | Numerator: medication-administration claims containing an antibacterial drug (ATC prefix J01). Denominator: all outpatient medication-administration claims. No additional exclusion was identified (p 3). | Evaluated by aggregate differential testing on real-world data. ATC J01 logic is aligned with the official rule; the denominator is currently visit based. | Denominator granularity differs (visit vs medication-administration claim). |
| 03-1 | Same-institution oral antihypertensive overlap (1710) | Numerator: overlapping medication-days from different prescriptions for the same patient at the same institution. Denominator: total medication-days. A chronic-prescription refill supplied for >=21 days and refilled within 10 days before prior supply exhaustion is exempt. Official ATC whitelist: C07 (excluding C07AA05), C02CA/C02DB/C02DC/C02DD, C03AA/C03BA/C03CA/C03DA, C08CA (excluding C08CA06), C08DA/C08DB, C09AA/C09CA; oral orders require eighth NHI-order-code digit=1 (p 3). | Evaluated by aggregate differential testing on real-world data. The current CQL uses broader ATC prefixes C02/C03/C07/C08/C09. The 10-day grace-period rule is now implemented and verified on synthetic controls. | ATC scope is broader than the official whitelist. Values reported for the final 88-patient cohort were computed with the corrected grace-period logic. |
| 03-2 | Same-institution oral lipid-lowering overlap (1711) | Numerator: overlapping medication-days. Denominator: medication-days for ATC C10AA/C10AB/C10AC/C10AD/C10AX with eighth NHI-order-code digit=1. The 10-day grace-period exemption applies (p 4). | Evaluated by aggregate differential testing on real-world data. CQL currently uses ATC prefix C10; the grace-period rule is implemented and synthetically verified. | Exact alignment with the official ATC whitelist was not evaluated in this study. Values reported for the final 88-patient cohort were computed with the corrected grace-period logic. |
| 03-3 | Same-institution glucose-lowering medication overlap (1712) | Numerator: overlapping medication-days. Denominator: medication-days for ATC A10AB/A10AC/A10AD/A10AE/A10BA/A10BB/A10BF/A10BG/A10BX/A10BH/A10BJ/A10BK, regardless of formulation. The 10-day grace-period exemption applies (p 4). | Evaluated by aggregate differential testing on real-world data. CQL currently uses ATC prefix A10; the grace-period rule is implemented and synthetically verified. | Exact alignment with the official ATC whitelist was not evaluated in this study. Values reported for the final 88-patient cohort were computed with the corrected grace-period logic. |
| 03-4 | Same-institution antipsychotic medication overlap (1726) | Numerator: overlapping medication-days. Denominator: medication-days for ATC N05AA/N05AB (excluding N05AB04)/N05AD/N05AE/N05AF/N05AH/N05AL/N05AN (excluding N05AN01)/N05AX/N05AC/N05AG. The 10-day grace-period exemption applies (p 5). | Evaluated by aggregate differential testing on real-world data. CQL currently uses ATC prefix N05A; the grace-period rule is implemented and synthetically verified. | The official list contains 2 explicit exclusions (N05AB04 and N05AN01); exact alignment with the official whitelist was not evaluated in this study. Values reported for the final 88-patient cohort were computed with the corrected grace-period logic. |
| 03-5 | Same-institution antidepressant medication overlap (1727) | Numerator: overlapping medication-days. Denominator: medication-days for ATC N06AA (excluding N06AA02 and N06AA12)/N06AB/N06AG. The 10-day grace-period exemption applies (p 5). | Evaluated by aggregate differential testing on real-world data. CQL currently uses ATC prefix N06A; the grace-period rule is implemented and synthetically verified. | Two official N06AA exclusions were not evaluated in this study. Values reported for the final 88-patient cohort were computed with the corrected grace-period logic. |
| 03-6 | Same-institution oral sedative-hypnotic overlap (1728) | Numerator: overlapping medication-days. Denominator: medication-days for ATC N05CC/N05CD/N05CF/N05CM with eighth NHI-order-code digit=1. The 10-day grace-period exemption applies (p 6). | Evaluated by aggregate differential testing on real-world data. CQL currently uses ATC prefix N05C; the grace-period rule is implemented and synthetically verified. | Exact alignment with the official ATC whitelist was not evaluated in this study. Values reported for the final 88-patient cohort were computed with the corrected grace-period logic. |
| 03-7 | Same-institution oral antithrombotic overlap (3375) | Numerator: overlapping medication-days; the official rule explicitly permits early collection of chronic refills. Denominator: medication-days for ATC B01AA/B01AC (excluding B01AC07)/B01AE/B01AF with eighth NHI-order-code digit=1. This indicator was added in 2023 (p 6). | Evaluated by aggregate differential testing on real-world data. CQL currently uses ATC prefix B01; the grace-period rule is implemented and synthetically verified. | The B01AC07 exclusion and exact alignment with the official whitelist were not evaluated in this study. Values reported for the final 88-patient cohort were computed with the corrected grace-period logic. |
| 03-8 | Same-institution oral benign-prostatic-hyperplasia medication overlap (3376) | Numerator: overlapping medication-days with the chronic-refill exemption. Denominator: medication-days for ATC G04CA/G04CB with eighth NHI-order-code digit=1 (p 7). | Evaluated by aggregate differential testing on real-world data. CQL currently uses ATC prefix G04C; the grace-period rule is implemented and synthetically verified. | Exact alignment with the official ATC whitelist was not evaluated in this study. Values reported for the final 88-patient cohort were computed with the corrected grace-period logic. |
| 03-9 | Cross-institution oral antihypertensive overlap (1713) | Numerator: cross-institution overlapping medication-days from different prescriptions for the same patient. Denominator: as in indicator 03-1. The 10-day grace-period exemption applies; cross-institution linkage is patient based (p 7). | Evaluated by aggregate differential testing on real-world data as a principal use case. Institutional boundaries are represented by Encounter.serviceProvider and the medical-institution identifier. The grace-period rule is implemented and synthetically verified. | ATC scope remains broader than the official whitelist. Values reported for the final 88-patient cohort were computed with the corrected grace-period logic. |
| 03-10 | Cross-institution oral lipid-lowering overlap (1714) | Numerator: cross-institution overlapping medication-days. Denominator: as in indicator 03-2. The 10-day grace-period exemption applies (p 8). | Evaluated by aggregate differential testing on real-world data as a principal use case; the grace-period rule is implemented and synthetically verified. | Exact alignment with the official ATC whitelist was not evaluated in this study. Values reported for the final 88-patient cohort were computed with the corrected grace-period logic. |
| 03-11 | Cross-institution glucose-lowering medication overlap (1715) | Numerator: cross-institution overlapping medication-days. Denominator: as in indicator 03-3. The 10-day grace-period exemption applies (p 8). | Evaluated by aggregate differential testing on real-world data as a principal use case; the grace-period rule is implemented and synthetically verified. | Exact alignment with the official ATC whitelist was not evaluated in this study. Values reported for the final 88-patient cohort were computed with the corrected grace-period logic. |
| 03-12 | Cross-institution antipsychotic medication overlap (1729) | Numerator: cross-institution overlapping medication-days. Denominator: as in indicator 03-4. The 10-day grace-period exemption applies (p 9). | Evaluated by aggregate differential testing on real-world data as a principal use case; the grace-period rule is implemented and synthetically verified. | The official ATC exclusions were not evaluated in this study. Values reported for the final 88-patient cohort were computed with the corrected grace-period logic. |
| 03-13 | Cross-institution antidepressant medication overlap (1730) | Numerator: cross-institution overlapping medication-days. Denominator: as in indicator 03-5. The 10-day grace-period exemption applies (p 9). | Evaluated by aggregate differential testing on real-world data as a principal use case; the grace-period rule is implemented and synthetically verified. | The official ATC exclusions were not evaluated in this study. Values reported for the final 88-patient cohort were computed with the corrected grace-period logic. |
| 03-14 | Cross-institution oral sedative-hypnotic overlap (1731) | Numerator: cross-institution overlapping medication-days. Denominator: as in indicator 03-6. The 10-day grace-period exemption applies (p 10). | Evaluated by aggregate differential testing on real-world data as a principal use case; the grace-period rule is implemented and synthetically verified. | Exact alignment with the official ATC whitelist was not evaluated in this study. Values reported for the final 88-patient cohort were computed with the corrected grace-period logic. |
| 03-15 | Cross-institution oral antithrombotic overlap (3377) | Numerator: cross-institution overlapping medication-days with the chronic-refill exemption. Denominator: as in indicator 03-7 (p 10). | Evaluated by aggregate differential testing on real-world data as a principal use case; the grace-period rule is implemented and synthetically verified. | The B01AC07 exclusion and exact ATC alignment were not evaluated in this study. Values reported for the final 88-patient cohort were computed with the corrected grace-period logic. |
| 03-16 | Cross-institution oral benign-prostatic-hyperplasia medication overlap (3378) | Numerator: cross-institution overlapping medication-days with the chronic-refill exemption. Denominator: as in indicator 03-8 (p 11). | Evaluated by aggregate differential testing on real-world data as a principal use case; the grace-period rule is implemented and synthetically verified. | Exact alignment with the official ATC whitelist was not evaluated in this study. Values reported for the final 88-patient cohort were computed with the corrected grace-period logic. |
| 04 | Chronic-disease continuous-prescription issuance rate (1318) | Numerator: chronic continuous-prescription claims identified by specified visit-fee codes or case category E1 with prescribed duration longer than dispensed days. Denominator: chronic-disease medication claims (case categories 04 and E1). Three hospital categories are excluded: obstetric/gynecologic specialty hospitals, pediatric specialty hospitals, and hospitals with >=80% respiratory-care-ward share (pp 11-12). | Evaluated by aggregate differential testing on real-world data. CQL uses a FHIR continuous-prescription marker as a proxy rather than the official visit-fee code list. | The denominator should be limited to chronic-disease case categories. The 3 hospital-category exclusions are not implemented; they do not affect the 2-campus cohort reported here. |
| 05 | Prescriptions with >=10 medication items (3128) | Numerator: denominator claims containing >=10 medication items. Denominator: medication-administration claims. Six numerator exclusions are specified: catastrophic illness, integrated home medical care, integrated care programs, emergency care, outpatient surgery, and STAT/PRN drugs (p 12). | Evaluated by aggregate differential testing on real-world data. CQL counts distinct medications within the same visit. | The 6 exclusions are not implemented individually; denominator granularity differs (medication-administration claim vs visit). |
| 06 | Emergency-department visit rate among patients with asthma aged <18 years (1315/1317) | Numerator: patients in the denominator with an asthma-related emergency visit, including cross-institution linkage. Denominator: patients satisfying any of 3 asthma definitions using emergency, inpatient, outpatient, medication, and prior-year national cross-institution data (p 13). One pathway requires >=4 outpatient asthma visits in the prior year with medication in >=2 visits. | Functionally verified on synthetic data only. Real-world evaluation awaits Condition, Encounter, and cross-year linkage data. | The multi-path denominator and prior-year linkage are complex; the current CQL is a single-observation-period approximation that was not evaluated against the official definition. |
| 07 | HbA1c or glycated-albumin testing rate among patients with diabetes | Numerator: denominator patients receiving HbA1c or glycated-albumin testing during the measurement period. Denominator: patients with an outpatient primary/secondary diabetes diagnosis (ICD-10-CM E08-E13) and a diabetes drug (ATC A10) in the same prescription claim (p 14). | Functionally verified on synthetic data only. | Strict same-prescription-claim linkage of diagnosis and medication was not evaluated at the indicator level. |
| 08 | Same-day, same-institution revisit for the same disease (1322) | Numerator: patients with >=2 visits in the same billing month, on the same day, at the same institution, and with the same first 3 characters of the primary diagnosis. Denominator: outpatient patients at the institution in the billing month. Ten shared exclusion groups cover cancer, copayment codes, case categories, short chronic refills, referral/testing cases, selected infections, and zero visit-fee claims (pp 14-15). | Functionally verified on synthetic data only. | The 10 exclusion groups are not implemented individually. |
| 09 | Unplanned readmission within 14 days (1077.01/1809) | Numerator: patients readmitted within 14 days. Denominator: quarterly discharges. Twelve planned-readmission exclusions cover psychiatry, cancer-related programs, pregnancy maintenance, mucopolysaccharidosis, transfers, newborn-card status, hemophilia, postcatheterization surgery, transplant, postacute care, and hospice care, using extensive NHI, ICD, and procedure code lists (pp 15-18). | Functionally verified on synthetic data only. | The 12 exclusion groups include extensive ICD-10-PCS code lists and are not implemented individually. |
| 10 | Emergency-department visit within 3 days after discharge (108.01) | Numerator: emergency visits within 3 days after discharge. Denominator: discharge claims. Ten exclusions cover psychiatry, cancer programs/treatment, pregnancy maintenance, mucopolysaccharidosis, transfers, newborn-card status, hemophilia, transplant, death, and discharge against medical advice while critically ill (pp 18-19). | Functionally verified on synthetic data only. | The 10 exclusions are not implemented individually. |
| 11-1 | Overall cesarean delivery rate (1136.01) | Numerator: cesarean delivery claims. Denominator: all deliveries (vaginal plus cesarean), identified through any of 3 mechanisms: first 3 digits of TW-DRG, DRG_CODE, or NHI order codes (pp 19-20). | Functionally verified on synthetic data only. | Completeness of the 3-part DRG/order-code identification standard was not evaluated in this study. |
| 11-2 | Cesarean delivery on maternal request (1137.01) | Numerator: cesarean delivery without an indication, identified by NHI order 97014C, DRG_CODE 0373B, or TW-DRG prefix 513. Denominator: all deliveries; the 3-part standard from indicator 11-1 applies (p 21). | Functionally verified on synthetic data only. | Same coding-completeness limitation as indicator 11-1. |
| 11-3 | Cesarean delivery with an indication (1138.01) | Numerator: cesarean delivery claims minus maternal-request cesarean claims. Denominator: all deliveries; the 3-part standard from indicator 11-1 applies (p 21). | Functionally verified on synthetic data only. | Same coding-completeness limitation as indicator 11-1. |
| 11-4 | Primary cesarean delivery with an indication (1075.01) | Numerator: first non–maternal-request cesarean delivery, identified by NHI orders 81004C/81028C or ICD-10-PCS 10D00Z0/Z1/Z2, excluding DRG 0373B and previous cesarean diagnosis O342. Denominator: all deliveries (p 22). | Functionally verified on synthetic data only. | The previous-cesarean exclusion is not implemented. |
| 12 | Antibiotic use for >3 days after clean surgery (1155) | Numerator: clean-surgery claims with postoperative antibiotic use for >3 days (ATC J01). Denominator: clean-surgery claims identified by case category 5 plus 3 surgery-code groups, excluding malignant neoplasm and selected infections (pp 22-23). Clean surgery requires combined ICD-10-PCS and NHI payment-standard codes. | Functionally verified on synthetic data only. CQL uses SNOMED CT clean-surgery codes and ATC J01C as an approximation. | The official dual ICD-10-PCS/payment-code definition differs from the CQL terminology; semantic correspondence was not evaluated in this study. |
| 13 | Mean extracorporeal shock-wave lithotripsy utilization (20.01/1804) | Numerator: number of extracorporeal shock-wave lithotripsy procedures. Denominator: patients receiving the procedure, identified by NHI orders 50023A-50026B. No special exclusion is specified (p 23). | Functionally verified on synthetic data only. | Mapping between NHI procedure codes and the CQL terminology was not evaluated in this study. |
| 14 | Readmission within 14 days after surgery for uterine fibroids (473.01) | Numerator: readmissions within 14 days for diagnoses related to the index surgery. Denominator: inpatient claims with a uterine-fibroid diagnosis (excluding cancer) and an excision/resection procedure. Diagnosis and procedure must occur in the same claim; related diagnoses are limited to ICD-10-CM N70-N85 (p 24). | Functionally verified on synthetic data only. | Strict same-claim linkage was not evaluated at the indicator level. |
| 15-1 | Infection within 90 days after total or hemi-knee arthroplasty (353.01) | Numerator: arthroplasty claims followed by a prosthesis-infection treatment order (64053B/64004C/64198B) within 90 days, including cross-institution events. Denominator: total- or hemi-knee arthroplasty claims (pp 24-25). | Functionally verified on synthetic data only. | Cross-institution infection linkage and the 90-day window were not evaluated in this study. |
| 15-2 | Infection within 90 days after total knee arthroplasty (3249) | Numerator: as in indicator 15-1. Denominator: total knee arthroplasty claims identified by NHI order 64164B. This is a reference indicator without a separate benchmark value (p 25). | Functionally verified on synthetic data only. | Same linkage and time-window limitation as indicator 15-1. |
| 15-3 | Infection within 90 days after hemi-knee arthroplasty (3250) | Numerator: as in indicator 15-1. Denominator: hemi-knee arthroplasty claims identified by NHI order 64169B. This is a reference indicator without a separate benchmark value (pp 25-26). | Functionally verified on synthetic data only. | Same linkage and time-window limitation as indicator 15-1. |
| 16 | Inpatient surgical wound infection rate (1658/1666) | Numerator: inpatients with a surgical wound infection. Denominator: all inpatients undergoing surgery, identified by 6-digit surgical order codes beginning 62-88 or 97. Wound infection is defined by an extensive ICD-10-CM diagnosis list (p 26). | Functionally verified on synthetic data only. | The extensive wound-infection diagnosis list has not been checked code by code against the CQL implementation. |
| 17 | Acute myocardial infarction mortality rate (1662/1668) | Numerator: deaths among denominator patients. Denominator: patients aged >=18 years with a principal acute myocardial infarction diagnosis (ICD-10-CM I21-I22). Death is identified from insurance death status or inpatient disposition code 4/A; transferred patients are excluded (p 27). | Functionally verified on synthetic data only; not validated on THAS or real institutional data. | Transfer exclusions and coverage of death sources in CQL were not evaluated in this study. |
| 18 | Palliative-care use among patients with dementia (2795/2796) | Numerator: denominator patients using palliative services. Denominator: patients with dementia (ICD-10-CM F01-F03/G30/G31 or specified full F10/F13/F18/F19 codes). Numerator services are limited to inpatient hospice care, shared hospice care, and home hospice order groups (pp 27-28). | Functionally verified on synthetic data only. | Mapping of the 3 official palliative-service order groups to CQL was not evaluated in this study. |
| 19 | Wound infection rate after clean surgery (2524/2526) | Numerator: patients undergoing clean surgery who develop a wound infection. Denominator: patients undergoing clean surgery, using the same clean-surgery definition as indicator 12. Wound infection uses a diagnosis-code set that differs from indicator 16 (pp 28-30). | Functionally verified on synthetic data only. | The official ICD-10-PCS plus payment-code definition was not evaluated against the CQL SNOMED CT approximation, as in indicator 12. |

# References

1. HL7 International. FHIR Release 4 (R4) specification. URL: https://hl7.org/fhir/R4/ [accessed 2026-07-19]

2. HL7 International. Clinical Quality Language (CQL) specification, release 1, STU 5. URL: https://cql.hl7.org/ [accessed 2026-07-19]

3. National Health Insurance Administration, Ministry of Health and Welfare (Taiwan). 2023 (Year 112) Hospital Global Budget Overall Medical Quality Information Disclosure Report, Professional Edition [in Chinese]. Taipei: NHIA; October 2024. URL: https://www.nhi.gov.tw/ch/dl-75209-eaaae0a444f5a4d379c6e1dd14e21929e-1.pdf [accessed 2026-07-18]
