# Supplementary material for: Implementing Taiwan National Health Insurance Quality Indicators as Clinical Quality Language on Fast Healthcare Interoperability Resources: Development and Feasibility Study
Source: JMIR Med Inform. 2026 Oct 15;14:e108093. doi: 10.2196/108093 (PMC13720404; doi:10.2196/108093)
Supplement: Supplementary file 4 [file medinform_v14i1e108093_app4.docx]

**Multimedia Appendix 4**

**Synthetic and Real-World Verification Results**

**Associated manuscript: Implementing Taiwan National Health Insurance Quality Indicators as Clinical Quality Language on FHIR: Development and Feasibility Study**

# Known-Answer Results for All 39 Indicators

The purpose-built synthetic verification set contained 1473 patients across FHIR R4 transaction bundles and 2 simulated hospital Organizations. Every indicator was evaluated against a fixed expected numerator and denominator. For indicators 03-1 to 03-16, numerator and denominator are expressed in drug-days; indicator 13 reports a mean utilization count. Identical values within the same-institution and cross-institution medication-overlap groups reflect the deliberate uniform branch-coverage quota, not a computational artifact.

| **Code** | **Indicator (NHI code)** | **Denominator unit** | **D** | **N** | **Rate** |
| --- | --- | --- | --- | --- | --- |
| 01 | Outpatient injection usage rate (3127) | Visits | 28 | 12 | 42.86% |
| 02 | Outpatient antibiotic usage rate (1140.01) | Visits | 28 | 14 | 50.00% |
| 03-1 | Same-institution antihypertensive overlap (1710) | Drug-days | 1512 | 101 | 6.68% |
| 03-2 | Same-institution lipid-lowering overlap (1711) | Drug-days | 1512 | 101 | 6.68% |
| 03-3 | Same-institution antidiabetic overlap (1712) | Drug-days | 1512 | 101 | 6.68% |
| 03-4 | Same-institution antipsychotic overlap (1726) | Drug-days | 1512 | 101 | 6.68% |
| 03-5 | Same-institution antidepressant overlap (1727) | Drug-days | 1512 | 101 | 6.68% |
| 03-6 | Same-institution sedative-hypnotic overlap (1728) | Drug-days | 1512 | 101 | 6.68% |
| 03-7 | Same-institution antithrombotic overlap (3375) | Drug-days | 1512 | 101 | 6.68% |
| 03-8 | Same-institution BPH overlap (3376) | Drug-days | 1512 | 101 | 6.68% |
| 03-9 | Cross-institution antihypertensive overlap (1713) | Drug-days | 1624 | 101 | 6.22% |
| 03-10 | Cross-institution lipid-lowering overlap (1714) | Drug-days | 1624 | 101 | 6.22% |
| 03-11 | Cross-institution antidiabetic overlap (1715) | Drug-days | 1624 | 101 | 6.22% |
| 03-12 | Cross-institution antipsychotic overlap (1729) | Drug-days | 1624 | 101 | 6.22% |
| 03-13 | Cross-institution antidepressant overlap (1730) | Drug-days | 1624 | 101 | 6.22% |
| 03-14 | Cross-institution sedative-hypnotic overlap (1731) | Drug-days | 1624 | 101 | 6.22% |
| 03-15 | Cross-institution antithrombotic overlap (3377) | Drug-days | 1624 | 101 | 6.22% |
| 03-16 | Cross-institution BPH overlap (3378) | Drug-days | 1624 | 101 | 6.22% |
| 04 | Chronic continuous prescription rate (1318) | Visits | 28 | 13 | 46.43% |
| 05 | Prescription ≥ 10 drug items rate (3128) | Visits | 26 | 12 | 46.15% |
| 06 | Pediatric (<18 y) asthma ED visit rate (1315/1317) | Patients | 20 | 8 | 40.00% |
| 07 | Diabetes HbA1c testing rate (109.01/110.01) | Patients | 25 | 10 | 40.00% |
| 08 | Same-day same-institution same-disease revisit (1322) | Visits | 30 | 8 | 26.67% |
| 09 | Unplanned 14-day readmission rate (1077.01/1809) | Patients | 60 | 13 | 21.67% |
| 10 | ED visit within 3 days of discharge (108.01) | Patients | 50 | 9 | 18.00% |
| 11-1 | Cesarean section rate — overall (1136.01) | Deliveries | 60 | 37 | 61.67% |
| 11-2 | Cesarean section — patient-requested (1137.01) | Deliveries | 60 | 11 | 18.33% |
| 11-3 | Cesarean section — with indication (1138.01) | Deliveries | 60 | 26 | 43.33% |
| 11-4 | Cesarean — first-time indicated (1075.01) | Deliveries | 60 | 20 | 33.33% |
| 12 | Clean surgery post-op antibiotics >3 days (1155) | Surgeries | 37 | 11 | 29.73% |
| 13 | ESWL average utilization (20.01/1804) | Patients | 35 | 63 | 1.80 (mean) |
| 14 | Uterine fibroid surgery 14-day readmission (473.01) | Surgeries | 35 | 7 | 20.00% |
| 15-1 | Knee arthroplasty 90-day deep infection (353.01) | Surgeries | 37 | 9 | 24.32% |
| 15-2 | Total knee arthroplasty 90-day infection (3249) | Surgeries | 22 | 6 | 27.27% |
| 15-3 | Partial knee arthroplasty 90-day infection (3250) | Surgeries | 15 | 3 | 20.00% |
| 16 | Inpatient surgical wound infection (1658/1666) | Surgeries | 55 | 12 | 21.82% |
| 17 | AMI mortality rate (1662/1668) | Patients | 55 | 8 | 14.55% |
| 18 | Dementia hospice utilization rate (2795/2796) | Patients | 65 | 17 | 26.15% |
| 19 | Clean surgery wound infection rate (2524/2526) | Surgeries | 55 | 12 | 21.82% |

# Chronic-Prescription Early-Refill Grace-Period Controls

For each of the 16 medication-overlap indicators, the synthetic verification set encodes 8 same-institution or 9 cross-institution control groups isolating the chronic-prescription (>=21-day supply) early-refill grace-period rule. All 8 same-institution indicators share the same control-group structure, as do all 8 cross-institution indicators. Representative results for indicators 03-1 and 03-9 are shown below. Across all 16 indicators, 136 control groups were evaluated and all matched their expected numerator and denominator classification (136/136).

## Same-institution representative—indicator 03-1 (antihypertensive), 8 control groups

| **Control group** | **n** | **Expected N** | **Engine N** | **Expected D** | **Engine D** | **Concordant** |
| --- | --- | --- | --- | --- | --- | --- |
| Positive control — acute overlap | 8 | 56 | 56 | 224 | 224 | Yes |
| Positive control — chronic overlap beyond window | 4 | 44 | 44 | 224 | 224 | Yes |
| Negative control — chronic exemption (grace period) | 8 | 0 | 0 | 448 | 448 | Yes |
| Boundary — refill 10 days before prior supply exhausted | 1 | 0 | 0 | 56 | 56 | Yes |
| Boundary — refill 11 days before prior supply exhausted | 1 | 1 | 1 | 56 | 56 | Yes |
| Within denominator — no overlap | 10 | 0 | 0 | 280 | 280 | Yes |
| Within denominator — single prescription | 8 | 0 | 0 | 224 | 224 | Yes |
| Denominator-excluded — non-oral formulation | 5 | 0 | 0 | 0 | 0 | Yes |

## Cross-institution representative—indicator 03-9 (antihypertensive), 9 control groups

| **Control group** | **n** | **Expected N** | **Engine N** | **Expected D** | **Engine D** | **Concordant** |
| --- | --- | --- | --- | --- | --- | --- |
| Positive control — acute overlap | 8 | 56 | 56 | 224 | 224 | Yes |
| Positive control — chronic overlap beyond window | 4 | 44 | 44 | 224 | 224 | Yes |
| Negative control — chronic exemption (grace period) | 8 | 0 | 0 | 448 | 448 | Yes |
| Boundary — refill 10 days before prior supply exhausted | 1 | 0 | 0 | 56 | 56 | Yes |
| Boundary — refill 11 days before prior supply exhausted | 1 | 1 | 1 | 56 | 56 | Yes |
| Within denominator — no overlap | 10 | 0 | 0 | 280 | 280 | Yes |
| Within denominator — single prescription | 8 | 0 | 0 | 224 | 224 | Yes |
| Negative control — same-institution overlap not counted as cross-institution | 2 | 0 | 0 | 112 | 112 | Yes |
| Denominator-excluded — non-oral formulation | 5 | 0 | 0 | 0 | 0 | Yes |

D=expected or engine denominator (drug-days); N=expected or engine numerator (drug-days). Identical structures apply to the remaining 14 medication-overlap indicators; full per-indicator control-group data are available in the study repository.

# Real-World Differential Testing

The final dataset included 88 patients (Taipei, n=65; Linkou, n=23), 1597 medication records, and 630 visits from January 1 to June 2, 2026. Pipeline A, an independent Python reference implementation, calculated each indicator directly from the source Excel medication long table. Pipeline B, the platform CQL engine, represented the same records as FHIR R4 Patient, Encounter, and MedicationRequest resources on a HAPI FHIR server and computed the medication indicators through the compiled-ELM pathway. Both numerator and denominator matched for all 20 computable indicators (20/20; 100%; descriptive exact 95% CI 83.2%-100%).

*Interpretation: this comparison evaluates agreement between the platform CQL engine and an independent reference implementation. It does not independently adjudicate the official clinical definition.*

**Table A2-4. Complete real-world differential-testing results (final 88-patient cohort).**

| **Code** | **Indicator** | **D (A)** | **N (A)** | **D (B)** | **N (B)** | **Rate** | **Concordant** |
| --- | --- | --- | --- | --- | --- | --- | --- |
| 01 | Outpatient injection usage | 630 | 16 | 630 | 16 | 2.54% | Yes |
| 02 | Outpatient antibiotic usage | 630 | 26 | 630 | 26 | 4.13% | Yes |
| 03-1 | Same-institution antihypertensive overlap | 5176 | 479 | 5176 | 479 | 9.25% | Yes |
| 03-2 | Same-institution lipid-lowering overlap | 3111 | 97 | 3111 | 97 | 3.12% | Yes |
| 03-3 | Same-institution antidiabetic overlap | 1658 | 130 | 1658 | 130 | 7.84% | Yes |
| 03-4 | Same-institution antipsychotic overlap | 1020 | 18 | 1020 | 18 | 1.76% | Yes |
| 03-5 | Same-institution antidepressant overlap | 502 | 7 | 502 | 7 | 1.39% | Yes |
| 03-6 | Same-institution sedative-hypnotic overlap | 1649 | 33 | 1649 | 33 | 2.00% | Yes |
| 03-7 | Same-institution antithrombotic overlap | 1148 | 43 | 1148 | 43 | 3.75% | Yes |
| 03-8 | Same-institution BPH overlap | 584 | 14 | 584 | 14 | 2.40% | Yes |
| 03-9 | Cross-institution antihypertensive overlap | 5176 | 50 | 5176 | 50 | 0.97% | Yes |
| 03-10 | Cross-institution lipid-lowering overlap | 3111 | 8 | 3111 | 8 | 0.26% | Yes |
| 03-11 | Cross-institution antidiabetic overlap | 1658 | 33 | 1658 | 33 | 1.99% | Yes |
| 03-12 | Cross-institution antipsychotic overlap | 1020 | 1 | 1020 | 1 | 0.10% | Yes |
| 03-13 | Cross-institution antidepressant overlap | 502 | 1 | 502 | 1 | 0.20% | Yes |
| 03-14 | Cross-institution sedative-hypnotic overlap | 1649 | 3 | 1649 | 3 | 0.18% | Yes |
| 03-15 | Cross-institution antithrombotic overlap | 1148 | 27 | 1148 | 27 | 2.35% | Yes |
| 03-16 | Cross-institution BPH overlap | 584 | 0 | 584 | 0 | 0.00% | Yes |
| 04 | Chronic continuous prescription rate | 630 | 342 | 630 | 342 | 54.29% | Yes |
| 05 | Prescription >=10 drug items rate | 630 | 0 | 630 | 0 | 0.00% | Yes |

D=denominator; N=numerator. For indicators 03-1 to 03-16, D and N are drug-days. For indicators 01, 02, 04, and 05, D and N are visits. Pipeline A=independent Python reference implementation computed from the source Excel long table; pipeline B=platform CQL engine computing from FHIR R4 resources through the compiled-ELM pathway. Rates are descriptive N/D percentages.

# References

1. HL7 International. Clinical Quality Language (CQL) specification, release 1, STU 5. URL: https://cql.hl7.org/ [accessed 2026-07-19]

2. National Health Insurance Administration, Ministry of Health and Welfare (Taiwan). 2023 (Year 112) Hospital Global Budget Overall Medical Quality Information Disclosure Report, Professional Edition [in Chinese]. Taipei: NHIA; October 2024. URL: https://www.nhi.gov.tw/ch/dl-75209-eaaae0a444f5a4d379c6e1dd14e21929e-1.pdf [accessed 2026-07-18]
